# Assessing Momentary Well-Being in People Living With Dementia: A Systematic Review of Observational Instruments

**DOI:** 10.3389/fpsyg.2021.742510

**Published:** 2021-11-23

**Authors:** Kristine Gustavsen Madsø, Elisabeth Flo-Groeneboom, Nancy A. Pachana, Inger Hilde Nordhus

**Affiliations:** ^1^Department of Clinical Psychology, Faculty of Psychology, University of Bergen, Bergen, Norway; ^2^NKS Olaviken Gerontopsychiatric Hospital, Bergen, Norway; ^3^School of Psychology, The University of Queensland, Brisbane, QLD, Australia; ^4^Department of Behavioral Medicine, Faculty of Medicine, University of Oslo, Oslo, Norway

**Keywords:** well-being, dementia, observation, emotion, systematic review, psychometric properties, engagement

## Abstract

Optimizing the possibility to lead good lives is at the core of treatment and care for people with dementia. This may be monitored by assessing well-being and quality of life. However, cognitive impairment following dementia may complicate recall-based assessment with questionnaires, and proxy-ratings from family-caregivers do not correspond well to self-reports. Thus, using observational measures represents a potentially advanced option. Systematic reviews evaluating measurement properties, interpretability and feasibility of observational instruments assessing well-being in people living with dementia are lacking. Thus, this review performed systematic searches to find peer reviewed validated instruments of relevance in the databases MEDLINE, EMBASE, PsycINFO, Web of Science, CINAHL and ProQuest. Twenty-two instruments assessing well-being were included for evaluation of measurement properties based on the systematic approach of the COnsensus-based Standards for the selection of health Measurement INstruments (COSMIN). The evaluation included risk of bias on study level, and assessment of measurement properties on instrument level including content validity, construct validity, structural validity, internal consistency, measurement invariance, cross-cultural validity, measurement error and inter-rater/intra-rater/test–retest reliability and responsiveness. Additionally, the feasibility and interpretability of the measures were evaluated. No single instrument could be recommended based on existing publications. Thus, we provide general recommendations about further assessment and development of these instruments. Finally, we describe the most promising instruments and offer guidance with respect to their implementation and use in clinical and research contexts.

## Introduction

Well-being and quality of life (QoL) are identified as core outcomes for psychosocial interventions by people living with dementia (Øksnebjerg et al., 2018; Reilly et al., 2020), in public health initiatives (World Health Organization [WHO], 2017), national guidelines (National Institute for Health and Care Excellence [NICE], 2018), and research recommendations (Dröes et al., 2016). World-wide, dementia is estimated to affect 50 million people (Livingston et al., 2017). Dementia is defined as a public health priority, causing disability and increasing dependency on help from others in the people affected (World Health Organization [WHO], 2017). However, increasing evidence highlights how people with dementia may live good lives in environments adapted to their physical, social, emotional, and psychological needs (Livingston et al., 2017).

To be able to ascertain whether the dementia care and interventions implemented actually promote individual well-being, valid measurement approaches reflecting well-being as it is described by the target group are needed (Madsø and Nordhus, 2021). In a recent scoping review, relevant well-being domains close to the experiences of people living with dementia were defined. These domains include positive emotions, experiencing meaning, a positive sense of self and a sense of agency, having rewarding relationships with significant others, and experiencing life satisfaction (Clarke et al., 2020). Well-being and QoL originate from separate research fields (Skevington and Böhnke, 2018), but have also been used synonymously in the dementia literature (Bowling et al., 2015). In this review, the term well-being is used when the domains are in line with Clarke et al. (2020).

In other populations, well-being is often measured by self-report (Ferring and Boll, 2010). It is well established that people in the earlier stages of dementia can provide valid self-reports of their well-being (Stoner et al., 2019; Clarke et al., 2020). Unfortunately, relying on self-report only may exclude people with more severe dementia, and reduce the possibility of longitudinal assessment throughout the degenerative course of the disease (Algar et al., 2016; Kaufmann and Engel, 2016). With increasing cognitive impairment, well-being is frequently assessed through *proxy-reports*. Proxy-reports refer to assessment of an individual based on the evaluations of informants other than the person themself. Studies have consistently found proxy-evaluations by family and professional caregivers to rate well-being lower as compared to self-reports (Sands et al., 2004; Kolanowski et al., 2007; Ferring and Boll, 2010; Schulz et al., 2013). The low correspondence between proxy-reports and self-report implies that well-being in dementia should be measured in face-to-face interviews for individuals able to give valid self-reports, together with observational measures by independent and neutral observers in those from whom self-reports may not be obtained (Ferring and Boll, 2010; Bowling et al., 2015).

It is well known that a measurement that relies on *retrospective* self-reports evaluating longer time-intervals is prone to bias because our autobiographical recall can be inaccurate and influenced by for example current mood (Shiffman et al., 2008). This may particularly be a source of bias in the dementia population due to impairments in memory, attention, insight, and communication skills (Ettema et al., 2007; Trigg et al., 2011). During retrospective self-report, the current emotional state may interfere with the judgment of the past (Kolanowski et al., 2014). Thus, the risk of substantial measurement error from self-reports is increased by the fluctuating nature of neuropsychiatric symptoms (Kales et al., 2015), as well as attention or awareness (Clare et al., 2012). Consequently, an alternative is to use Ecological Momentary Assessment (EMA) and assess well-being within a momentary timeframe that can detect clinically relevant variations occurring over short time intervals (Shiffman et al., 2008). EMA consists of several approaches - direct observation is one of them. Assessing well-being in dementia through observing behavior as it occurs is one approach that can omit several of the problems and sources of bias related to measurement in dementia as mentioned above (Ferring and Boll, 2010). In sum, observational methods are advantageous because (1) they can be used to assess subjects that struggle with self-report, (2) neutral observers may provide more accurate evaluations than proxies, (3) it is not dependent on memory of the past, and (4) it is sensitive to changes in state.

However, the well-being domains identified as central in dementia by Clarke et al. (2020) are not all available for assessment through observation. Assessing well-being through observation implies coding or rating behavioral expressions, bodily positions, verbal or non-verbal expressions, or facial expressions that are all assumed to indicate the inner state of the observed person. Thus, we suggest observable aspects in line with the model of Clarke et al. (2020) are operationalized expressions of well-being in terms of positive behavioral expressions, balance between positive and negative emotions, level of engagement, expressions of satisfaction, and quality of social relationships. These aspects reflect central domains from the perspective of people living with dementia (Clarke et al., 2020) and central theories of well-being (Diener, 1984) and well-being in dementia (Lawton et al., 1996; Kitwood, 1997). The remaining domains of Clarke et al. (2020) related to experiencing meaning, having a positive sense of self and a sense of agency, may better be assessed through self-report. Still, accessing these domains and describing them may be difficult for people with more moderate and severe dementia.

Former reviews have reported on a variety of observational measures for people living with dementia (Curyto et al., 2008), including observational instruments specific for well-being and QoL in dementia (Algar et al., 2016), and measurements of emotional expressions in dementia (Lee et al., 2019). However, there is a lack of systematic reviews evaluating measurement instruments assessing momentary well-being in dementia that includes an evaluation against quality criteria and risk of bias. The COnsensus-based Standards for the selection of health Measurement INstruments (COSMIN) initiative is a relevant systematic approach for reviewing health related outcome instruments (Prinsen et al., 2018). COSMIN is developed through extensive Delphi-studies with experts and in concordance with well-established systematic approaches for conducting reviews such as the Cochrane Handbook, the PRISMA statement, and the Grading of Recommendations Assessment, Development and Evaluation (GRADE) principles (Mokkink et al., 2017; Prinsen et al., 2018; Terwee et al., 2018).

Our objective is to systematically review the literature and inform researchers and practitioners about the current state of knowledge and clinical utility of observational instruments assessing momentary well-being, to support care and interventions for people living with dementia. Guided by the COSMIN-framework, this systematic review aims to:

1. Identify observational instruments assessing momentary well-being in people with dementia.
2. Evaluate study specific methodological quality of the included publications through risk of bias (RoB) ratings.
3. Evaluate and compare measurement properties against quality criteria at instrument level.
4. Summarize and grade the trustworthiness of the body of evidence for each instrument.
5. Assess feasibility and interpretability of the instruments.

## Methods

**FIGURE 1:**
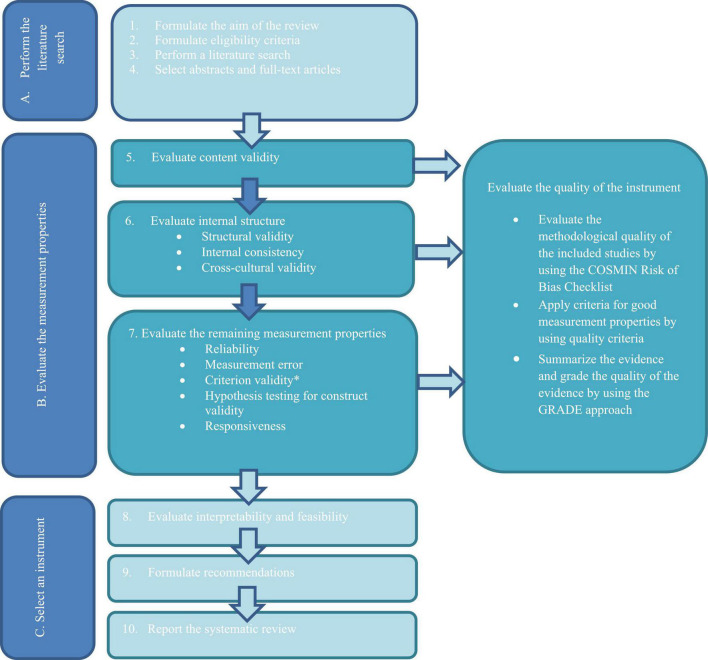
COnsensus-based Standards for the selection of health Measurement INstruments (COSMIN) guideline for systematic reviews of health-related measurement instruments. Reprint of this figure from Prinsen et al. (2018) is permitted under the Creative Commons Attribution 4.0. (http://creativecommons.org/licenses/by/4.0/). The acronym PROM (Patient Rated Outcome Instrument) is changed to “instrument” in this reprint. *Criterion validity was not assessed in this review, as no gold-standard instrument for comparison was identified.

The protocol for this review was pre-registered in the international register of systematic reviews, PROSPERO (RRID:SCR_019061, ID: 176160). Figure 1 describes the COSMIN-guideline for conducting systematic reviews on health-related outcome measures that was utilized in this review.

### Inclusion and Exclusion Criteria

Criteria for inclusion were (a) observational measures of momentary well-being (b) assessed by independent observers (c) during direct observation or video-recordings, containing (d) observable operationalizations of well-being such as positive and negative emotions/affect, or behavioral displays of satisfaction or engagement. Instruments should assess well-being (e) before, during and/or after psychosocial interventions over (f) short time intervals (minutes or hours). At least one psychometric property should be reported, and g) instruments not exclusively assessing well-being could be included, but only the well-being domain would be assessed. Instruments developed for the general population could be included if they also were specifically tested in people with dementia. Only English peer-reviewed journal articles were included.

Exclusion criteria were observational instruments (a) focusing merely on ill-being, such as negative emotions, anxiety, depression or neuropsychiatric symptoms, and instruments measuring (b) observable physiological indicators of well-being only (such as biomarkers or startle reflex).

### Search Strategy

Searches were performed on April 21st, 2020, and repeated on April 06th, 2021, in the databases MEDLINE, EMBASE, PsycINFO (all via OVD), Web of Science^TM^, CINAHL (via EBSCOhost) and ProQuest^®^ (Psychology and Nursing and Allied Health). A combination of the words “well-being,” “dementia,” “observation,” “measurement,” and “psychometric properties” were searched for, using both Boolean operators and truncations. We utilized the published search filter with words describing measurement properties of outcome instruments from Terwee et al. (2009). The full search strategy corresponding to the databases is available in Supplementary Appendix A.

The search was limited to peer-reviewed journal articles, searching in title, abstract and subject headings. In addition, we hand-searched reference lists of relevant reviews, investigated reference lists and forward chained citations of the included publications. Authors of relevant articles were contacted when the publication did not provide the full observation tool. Other publication types, such as conference proceedings, editorials and books were excluded (Prinsen et al., 2018), as were articles where the instrument was not accessible and lacked a full description of the operationalizations of the items in the publication.

### Selection of Studies

The first author (KM) carried out the searches in the databases, imported the results to Endnote^®^ (RRID:SCR_014001) where the results were checked, and duplicates removed. Next, KGM screened the titles and imported the records eligible for screening of abstract to Rayyan QCRI^®^ (RRID:SCR_017584). KM also conducted hand searches of relevant records and imported these to Rayyan. The first (KM) and last (IN) author independently screened the records in Rayyan based on the eligibility criteria. Next, the results from the independent screening were compared, and all conflicts and their solutions of abstract screening were logged to ensure transparency. The next step was to evaluate the included publication based on full text. KM and IN read the full text independently and evaluated the publications against eligibility criteria in team meetings.

### Data Extraction

Extraction was conducted by the first author (KM) and reviewed by a team including three of the authors (KM, EF-G, and IN). 20% of the data was extracted twice by the first author (KM) to ensue correct extraction. The extraction procedure was predefined and based on the COSMIN extraction tables (Prinsen et al., 2018). The first category addressed conceptualization (overarching conceptualization of well-being, population the instrument was developed in, and well-being domains assessed). The second category addressed central study characteristics (population, setting, methods, and results) for publications reporting on any of the measurement properties “content validity,” “structural validity,” “internal consistency,” “cross-cultural validity/measurement invariance,” “reliability,” “measurement error,” “construct validity” through hypothesis testing, and “responsiveness” (Mokkink et al., 2017; Prinsen et al., 2018; Terwee et al., 2018). The third category addressed feasibility (procedure, granularity, concreteness, training, requirements) and interpretability (measurement level and scoring, primary recording units, distribution, and sensitivity; Bakeman and Quera, 2012; Mokkink et al., 2017; Prinsen et al., 2018; Terwee et al., 2018).

Granularity refers to how fine grained and detailed the instrument is. Concreteness refers to how physically based the items are, where high concreteness involves bodily movement and low concreteness allows for interpretation of inner states. Measurement level defines which research questions may be asked, from nominal and ordinal to continuous output. Lastly, the primary recording unit defines how you sample the observations, from counting specific events in continuous or pre-specified intervals, to continuous recordings of duration (Bakeman and Quera, 2012; Chorney et al., 2015).

An overview of the COSMIN-definitions of central measurement properties of health-related instruments are provided in Table 1.

**TABLE 1:** COnsensus-based Standards for the selection of health Measurement INstruments (COSMIN) definitions of central terms.

Term	Definition^1^
***Validity***	*The degree to which an instrument measures the construct(s) it purports to measure*
*Content validity*	*The degree to which the content of an instrument is an adequate reflection of the construct(s) it purports to measure*
*Construct validity^ab^*	*The degree to which the scores of an instrument is consistent with hypotheses (for instance with regards to internal relationships to scores of other instruments, or differences between relevant groups) based on the assumption that the instrument validly measures the construct to be measured*
*Structural validity^c^*	*The degree to which the scores of an instrument are an adequate reflection of the dimensionality of the construct to be measured*
*Cross-cultural validity*	*The degree to which the performance of the items on a translated or culturally adapted instrument are an adequate reflection of the performance of the items of the original version of the instrument*
*Measurement invariance^2^*	*Whether respondents from different groups with the same latent trait level (allowing for group differences) respond similarly to a particular item*
***Reliability (extended definition)***	*The extent to which scores for patients who have not changed are the same for repeated measurement under several conditions: e.g., using different sets of items from the same* [instrument] *(internal consistency); over time (test–retest); by different persons on the same occasion (inter-rater); or by the same persons (i.e., raters or responders) on different occasions (intra-rater)*
*Internal consistency*	*The degree of the interrelatedness among the items*
*Measurement error*	*The systematic and random error of a patient’s score that is not attributed to true changes in the construct to be measured*
*Reliability*	*The proportion of the total variance in the measurement which is due to “true” differences between patients*
***Responsiveness* ^b^**	*The ability of an instrument to detect change over time in the construct to be measured*
***Interpretability***	*Interpretability is the degree to which one can assign qualitative meaning – that is, clinical or commonly understood connotations – to an instruments quantitative scores or change in scores*

*^1^Reprint of definitions permitted by the COSMIN-initiative. Original definitions are written in *italics*, and changes as regular text. (by the COSMIN team, all but, ^2^ available at https://cosmin.nl/wp-content/uploads/COSMIN-definitions-domains-measurement-properties.pdf.*

*^2^ available at p. 51 https://cosmin.nl/wp-content/uploads/COSMIN-syst-review-for-PROMs-manual_version-1_feb-2018.pdf.*

*^*a*^As no gold standard for observing well-being in the field of dementia could be identified (Algar et al., 2016), *criterion validity* could not be evaluated (Prinsen et al., 2018). In this case, guidelines recommend to evaluate comparisons with other instruments as *hypotheses testing for construct validity* (Mokkink et al., 2017). These may be reported in the original publication as criterion validity, concurrent validity, convergent or divergent validity.*

*^*b*^While construct validity concerns hypothesis of correlations of *single scores* of similar instrument, responsiveness concerns testing hypotheses of correlations of *change-scores* of similar instruments to investigate the instruments ability to detect change (de Vet et al., 2011).*

*^*c*^ In COSMIN, distinctions are made between *reflective* and *formative* instruments (de Vet et al., 2011). Reflective instruments (or subscales) are unidimensional, where increase in any item reflects an increase in the construct of interest. The evaluation of structural validity and internal consistency is only relevant for reflective scales with more than one item. Structural validity is the investigation of the expected unidimensionality of the instrument, and internal consistency is investigating the expected correlations between the items. Formative models have multidimensional structure and items may cause or form the construct independent of each other (de Vet et al., 2011).*

### Evaluating Methodological Quality

Study specific RoB-ratings from multiple sources per instrument were ranked with the categories “very good,” “adequate,” “doubtful,” “inadequate,” and “not applicable.” RoB-ratings were conducted by KM and IN in collaboration. Conflicting ratings were discussed with EF-G or NP. Rating criteria were based on the COSMIN RoB Checklist (Mokkink et al., 2017; Prinsen et al., 2018). The COSMIN-framework is created for patient-reported measurement instruments. To fit the COSMIN evaluations to the specific requirements for observational measures, some adaptations to the COSMIN-criteria were necessary. These mainly regarded the evaluation of content validity of the instruments. Our adaptations were based on recommendations from Bakeman and Quera (2011) and Bakeman and Quera (2012), and can be found in the Supplementary Material (Supplementary Table 1).

Consensus-based Standards for selection of health Measurement Instruments (COSMIN)-criteria for the *content validity* of self-reported measures are strongly based on feedback from the target group to assess relevance, comprehensiveness, and comprehensibility of the content of an instrument. Criteria for “relevance” requires items to be relevant for the construct of interest, the target population, and the context of use. To be “comprehensive,” the items need to cover all key aspects of the construct (Terwee et al., 2018). We adapted the evaluations of content validity to observational measures based on Bakeman and Quera (2012); Chorney et al. (2015); and Perugia et al. (2018b). To get an “adequate” or “good” rating of content validity, our team decided at least two of the following approaches were required: theoretical approaches with literature reviews, qualitative field work and development of coding scheme or ethogram, and quantitative survey or qualitative interviews including the target group (people with dementia or their close care givers and/or experts from all relevant disciplines). In addition, lack of pilot field testing followed by evaluation and revision of the “comprehensibility” of the instrument lead to a rating of “inadequate.”

Content validity is context- and population specific, implying that in this review the instruments’ content validity is evaluated for the specific construct (well-being) in the specific context of evaluating psychosocial interventions for persons living with dementia (Terwee et al., 2018). Thus, evidence of content validity in other populations or contexts may not be generalizable and are not included.

As lack of *a priori* hypotheses is a common bias in health-related measurement development, we used a recommended generic hypothesis from COSMIN for evaluating construct validity and responsiveness (Prinsen et al., 2018, Table 4, p. 1154). COSMIN recommends *similar* constructs to be evaluated against a threshold of ± ≥ 0.5, and r*elated but dissimilar* constructs to be evaluated against a threshold of ± ≥ 0.3. Defining constructs as similar or only related *a priori* is a complex task. Relevant sources of measurement error identified in previous reviews are: (1) comparisons between state or trait dimensions (Curyto et al., 2008); (2) comparing self-, proxy- and observer-rated measures (Ferring and Boll, 2010); and (3) comparing instruments with different timeframes (Shiffman et al., 2008). Thus, we chose to use the recommended threshold of ± ≥ 0.3 as our threshold of comparison.

In addition, we did not expect decreasing well-being-scores to correlate with increasing dementia severity or cognitive impairment, as these constructs are found to be independent in several reviews (e.g., Missotten et al., 2008; Martyr et al., 2018).

Inter-rater reliability and agreement are particularly important properties of observational measures, and the new COSMIN-consensus regarding ratings of reliability and measurement error for clinician rated instruments was incorporated (Mokkink et al., 2020). The principle for overall quality scorings is ‘the worst score counts’, and one uses the lowest rating of the measurement property to indicate RoB (Mokkink et al., 2017; Prinsen et al., 2018; Terwee et al., 2018). COSMIN guidelines are available at www.cosmin.nl.

### Data Synthesis

After the initial study specific evaluation, the total evidence provided for each instrument was rated against adapted COSMIN quality criteria using the ratings “good” (+), “unclear” (?), “inadequate” (-), = “conflicting” (±), “not evaluated” (NE), and “not applicable” (NA). Table 2 provides an overview of the quality criteria. As most instruments were investigated in one publication only, no quantitative data synthesis was obtainable except for construct validity. For construct validity, the summarized number of hypotheses supporting the construct was divided by the sum of hypotheses (Prinsen et al., 2018).

**TABLE 2:** Adapted COnsensus-based Standards for the selection of health Measurement INstruments (COSMIN)-quality criteria.

Property	Rating	Criteria
Content validity^a^	+	Both total relevance and comprehensiveness is rated as ‘ + ’ and development study is not rated as ‘inadequate.’ An appropriate quantitative or qualitative data collection method used to identify relevant and comprehensive items for the instrument. At least two approaches used: theoretical approach with literature review, adaptations of other coding schemes, qualitative field work and development of coding scheme or ethogram, quantitative survey or qualitative interviews and focus groups including target group (experts from all relevant disciplines and/or patients and family care givers). Pilot test conducted. If there is a lack of evidence, the evaluation of the reviewers will determine overall rating
	−	Both total scores of relevance and comprehensiveness is rated ‘-’
	±	One of the two scores of relevance and comprehensiveness is rated ‘-’ and the other is rated ‘ + ’
Structural validity^b^	+	***CTT***: *CFA: CFI or TLI or comparable measure* > *0.95 OR RMSEA* < *0.06 OR SRMR* < *0.08* ***IRT/Rasch***: *No violation of unidimensionality: CFI or TLI or comparable measure* > *0.95 OR RMSEA* < *0.06 OR SRMR* < *0.08 AND no violation of local independence: residual correlations among the items after controlling for the dominant factor* < *0.20 OR Q3’s* < *0.37 AND no violation of monotonicity: adequate looking graphs OR item scalability* > *0.30 AND adequate model fit IRT:*χ*^2^* > *0.001 Rasch: infit and outfit mean squares* ≥ *0.5 and* ≤ *1.5 OR Z-standardized values* > −*2 and* < *2*
	?	***CTT***: *not all information for* ‘ + ’ *reported* ***IRT/Rasch****: model fit not reported*
	−	*Criteria for* ‘ + ’ *not met*
Internal consistency^b^	+	*At least low evidence for sufficient structural validity AND Cronbach’s alpha(s)* ≥ *0.70 for each unidimensional scale or subscale*
	?	*Criteria for “At least low evidence for sufficient structural validity” not met*
	−	*At least low evidence for sufficient structural validity AND Cronbach’s alpha(s)* < *0.70 for each unidimensional scale or subscale*
Reliability^c^	+	*For continuous scores: ICC* ≥ *0.70 For ordinal or nominal scores: (weighted) Kappa* ≥ *0.70*
	?	*ICC or (weighted) Kappa not reported*
	−	*ICC or (weighted) Kappa* < *0.70*
Measurement error^c^	+	For continuous scores: SDC or LoA or CV*√2*0.196 < *M(C)IC For ordinal/nominal/dichotomous scores: Percentage specific (e.g., positive and negative) agreement calculated and above 80%*
	?	*MIC not defined*
	−	For continuous scores: SDC or LoA or CV*√2*0.196 > *M(C)IC For ordinal/nominal/dichotomous scores: Percentage specific (e.g., positive and negative) agreement calculated and above 80%*
Hypotheses-testing for construct validity^b^	+	*The results are in accordance with* > *75% of the hypotheses*, and correlations with similar instruments are > 0.3
	?	Unclear hypotheses
	±	Results are in accordance with less than 75% of the hypotheses
	−	*The result is not in accordance with the hypotheses*, or all correlations are below >. 3
Cross-cultural validity/measurement invariance^b^	+	*No important differences found between group factors (such as age, gender, language) in multiple group factor analysis OR no important DIF for group factors (McFadden’s R^2^* < *0.02)*
	?	*No multiple group factor analysis OR DIF analysis performed*
	−	*Important differences between group factors OR DIF was found*
Responsiveness^b^	+	*The result is in accordance with* > *75% of the hypotheses, OR AUC* ≥ *0.70*
	?	Unclear hypotheses
	±	Results are in accordance with less than 75% of the hypotheses
	−	*The result is not in accordance with the hypotheses, OR AUC* < *0.70*

*^*a*^Criteria is adapted from Terwee et al. (2018), available in the following COSMIN-manual (pp 58-59) https://cosmin.nl/wp-content/uploads/COSMIN-methodology-for-content-validity-user-manual-v1.pdf. Adaptations based on specific recommendations for development of observational instruments from Bakeman and Quera (2012).*

*^*b*^Criteria from Prinsen et al. (2018, p. 1152).*

*^*c*^Criteria from Mokkink et al. (2020) available in the following COSMIN-manual (p. 55) https://www.cosmin.nl/wp-content/uploads/user-manual-COSMIN-Risk-of-Bias-tool_v4_JAN_final.pdf. Reprint of tables from these three sources are permitted under the Creative Commons Attribution 4.0 (http://creativecommons.org/licenses/by/4.0/). Original criteria are written in *italics*, our adaptations are written as regular text. ***Abbreviations***: AUC, Area under the curve; CFA, confirmatory factor analysis; CFI, comparative fit index; CTT, classical test theory; CV, Coefficient of Variation; DIF, differential item functioning; ICC, intraclass correlation coefficient; IRT, Item response theory; LoA, Limits of Agreement; MIC, minimal important change; RMSEA, Root Mean Square Error of Approximation; SDC, Smallest detectable change; SRMR, Standardized Root Mean Residuals; TLI, Tucker-Lewis index. ***Ratings:*** +, good; ?, unclear; −, inadequate; ±, conflicting; NE, not evaluated, NA, not applicable. Structural validity or internal consistency is reported as “not applicable” for instruments evaluated as formative.*

The trustworthiness of the summarized quality criteria rating was ranked with Grading of Recommendations Assessment, Development and Evaluation (GRADE) principles (GRADE Handbook, 2013), modified in the COSMIN approach for the context of health-related outcome measures (Prinsen et al., 2018). Four factors are assessed on instrument level: “risk of bias,” “inconsistency,” “imprecision,” and “indirectness” of the evidence, graded as “high,” “moderate,” “low,” or “very low”. Ratings were conducted in team meetings with KM and IN, including EF-G if consensus was not met.

## Results

### Search Results

**FIGURE 2:**
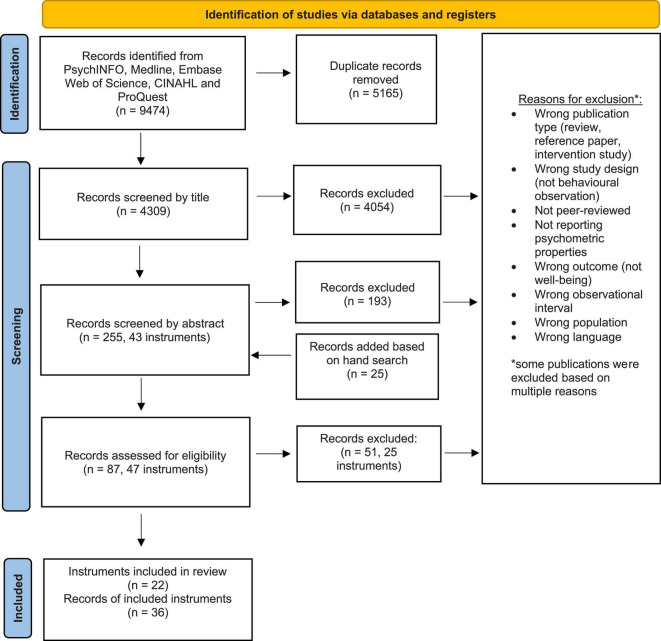
PRISMA Flow chart of search results.

Search results and reasons for exclusion is presented in Figure 2. After removing duplicates, KM screened 4309 records by title. Then, the 255 publications eligible for evaluation of abstracts was blind screened for inclusion by KM and IN (82% agreement). Additionally, 25 publications were added through hand search of relevant records. After full-text review of 87 records by KM and IN, 36 articles describing a total of 22 instruments were included, of which three originated from the hand-search.

### Conceptualizations of the Included Instruments

Key characteristics of the instrument, target population and domains are presented in Table 3. The included instruments are sorted in the three (not mutually exclusive) categories (a) observations of emotions, (b) observations of positive behavioral expressions, and (c) observations of engagement. Instruments are presented in chronological order within each category.

**TABLE 3:** Characteristics of the included instruments.

Key references	Target population	Items/domains
**EMOTIONS**		
***(Emotion) Facial Action Coding System (EMFACS/FACS)*** – describing positive and negative emotions based on		
facial behavior through action units (FACS) or systematic combination of action units expressing emotions (EMFACS)		

Ekman and Friesen (1978), Asplund et al. (1991), Asplund et al. (1995)	Generic instrument. Tested in people with moderate to severe dementia.	FACS – 27 descriptive action units EMFACS – combination of action units as emotions. Items tested in dementia research: Joy, surprise, sadness, anger, fear, disgust, and contempt

**The Maximally Discriminative Facial Movement Coding System (MAX)** – observing facial expressions of primary emotions		

Izard (1979), Izard (1995), Magai et al. (1996)	Generic instrument. Tested in persons with moderate to severe dementia.	13 descriptive units of facial behavior in mouth-lip region, 8 units in eyes-nose-cheek region, 6 units in brow region Formulas determine if one of eight emotions are detected: Joy, sadness, fear, anger, surprise, disgust, contempt, and interest

**Observed Emotion Rating Scale (OERS)** - Assessing emotions experienced by persons with Alzheimer’s dementia		

Lawton et al. (1999)	Moderate to severe Alzheimer’s dementia	Positive affect: Pleasure and interest Negative affect: Anger, anxiety/fear, and depression/sadness

**The Apparent Emotion Rating Scale (AER)** - Assessing positive and negative affect in geriatric populations.		

Snyder et al. (1998)	Geriatric populations with and without cognitive impairment in nursing homes, adult day care and research settings	Positive affect: Pleasure, interest, and tranquility Negative affect: Sadness, anxiety, and anger 15 verbal or non-verbal indicators for each domain.

**Observable Displays of Affect (ODAS)** - Behavioral displays of positive and negative affect following interventions		

Vogelpohl and Beck (1997), Beck et al. (2002)	People with dementia in nursing homes	41 behaviors of positive and negative affect categorized in six subscales 1. Facial positive displays 2. Facial negative displays 3. Vocal positive displays 4. Vocal negative displays 5. Body positive movement/posture 6. Body negative movement/posture

**POSITIVE EXPRESSIONS**		

**Dementia Care Mapping version 8 (DCM-8)** - Assessing psychological well-being and the quality of care in people with dementia in care settings		

Bradford Dementia Group (2005), Brooker and Surr (2006)	People with dementia in care settings	Combinations of Mood and Engagement (MEs) scores in correspondence to co-occurring Behavior Category Codes (BCCs) Additional: Personal Enhancers, Personal Detractions, and contextual field notes

**Positive Response Schedule (PRS)** – Assessing well-being in people with dementia through understanding occupational needs		

Perrin (1997)	People severely impaired by dementia	10 behavioral categories: Deliberate body movement, deliberate head movement, vocalization, looks at environment, looks at carer, initiates interaction, engagement, happy, sad, and fear

**Activity in Context and Time (ACT) –** Assessing environmental correlates of daily patterns of time use and well-being		

Wood (2005)	People with dementia in long term care settings	Environmental context domains (activity, social and physical) coded in relation to time use domains (positive behavior; gaze, mobility, conversation, and activity, negative behavior; agitation) and apparent affect (positive, negative, or null affect). Corresponding modifiers are created for each domain.

**Greater Cincinnati Chapter Well-Being Observational Tool (GCWBT)** – Assessing psychological well-being in people with dementia		

Kinney and Rentz (2005)	People with dementia in adult day programs, assisted living and long-term care. Assessing creative art interventions	Seven domains with 19 indicators of well-being: interest, sustained attention, pleasure, negative affect, sadness, self-esteem, and normalcy

**Scripps Modified Greater Cincinnati Chapter Well-being Observation Tool (SM-GCWBT**) - Psychological well-being and ill-being in people with dementia		

Sauer et al. (2016), Lokon et al. (2019)	Persons with moderate to advanced dementia in creative art interventions.	Two domains with 25 indicators Well-being: social interest, engagement, pleasure Ill-being: disengagement, negative affect, sadness, and confusion Domains scored on both frequency and intensity

**AwareCare** – Assessing behavioral signs of awareness and response to stimuli in people with severe dementia		

Clare et al. (2012)	People with severe dementia in care settings	10 different stimuli (not reviewed here) and 14 response categories: Eyes: eyes flicker, makes eye contact, explores with eyes Face: smiles, frowns, nods/shakes, moves head Limbs: reaches, grasps/holds Body: moves toward, moves away Vocalizations: single words, mumbles, shouts/moans

**Behavior, Engagement and Affect Measure (BEAM) -** Behavioral agitation, engagement and affect in people with dementia		

Casey et al. (2014)	People with mild to severe dementia living in long term care	Nine domains - Mobility status, activity context, agitation, positive behavior, engagement, affect, interaction: initiator, interaction: recipient, global contentment

**Maastricht Electronic Daily Life Observational tool (MEDLO-tool)** - Daily life aspects in long-term care, including emotional wellbeing		

de Boer et al. (2016)	Nursing home residents with moderate to severe dementia	Four domains: activity, physical environment, social interaction, and emotional well-being.

**COMMUNI-CARE** – Assessing psycho-emotional well-being in persons with dementia		

Lopez et al. (2016)	People with moderate to severe dementia during multi-sensory Snoezelen interventions	Five items – anxiety, communication, pleasure, adaptation to the surroundings, and affection

**QUALIDEM for intensive longitudinal assessment (QUALIDEM-ILA)** – Assessing momentary well-being of life in people with dementia		

Junge et al. (2020)	People with mild to severe dementia living in nursing homes.	Short version of QUALIDEM (Ettema et al., 2007) with 8 items in the following domains: restlessness, mood, anxiousness, body language, communication, happiness, sadness, and sociability

**ENGAGEMENT**		

**Menorah Park Engagement Scale (MPES)** - Engagement in activities		

Judge et al. (2000)	People with dementia in day care settings	Four categories of engagement: constructive engagement, passive engagement, non-engagement, and self-engagement

**Observational measurement of Engagement (OME)** - Engagement toward stimulus in persons with dementia		

Cohen-Mansfield et al. (2009)	People with dementia in long term care	Observations of response to stimuli: rate of refusal, duration of interest, attention, attitude, and activity

**Music in Dementia Assessment Scales (MiDAS)** - Musical engagement in music therapy for people with dementia		

McDermott et al. (2014), McDermott et al. (2015)	People with moderate to severe dementia receiving music therapy	Five visual analog subscales: interest, response, initiation, involvement, enjoyment Supplementary checklist of notable reactions during assessment (agitation/aggression, withdrawn/low in mood, restless/anxious, relaxed mood, attentive/interested, cheerful/smiling)

**Video Coding – Incorporating Observed Emotion (VC-IOE)** - Engagement toward stimulus (social robots)		

Jones et al. (2015)	People with dementia in care-settings	Six engagement-types with mutually exclusive operationalizations: emotion, verbal engagement, visual engagement, behavioral engagement, collective engagement, and agitation

**Engagement of a Person with Dementia Scale (EPWDS) -** Engagement toward an activity		

Jones et al. (2018)	People with dementia in acute, community and long-term care	Positive engagement or disengagement/negative engagement in the following five dimensions: affect, visual, verbal, behavioral and social

**Ethographic and Laban Inspired Coding System of Engagement (ELICSE)** and **Evidence-Based Model of Engagement-Related Behavior (EMODEB)**		
– Engagement naturally expressed through behaviors in activities of game-based and robot-based play		

Perugia et al. (2018b)	Mild to moderately severe dementia, nursing homes	13 different behaviors in three body parts. Head behavior, torso behavior and arms/hands behavior, and their following affective gestural support

**Music therapy engagement scale (MTED)** - Engagement in music therapy		

Tan et al. (2019)	Persons with dementia in acute hospital settings	Five domains of engagement: musical engagement, relatedness through music, verbal communication, emotional responsiveness, and overall responsiveness

#### Observations of Emotions

Five instruments were identified assessing emotion through operationalizations of facial, bodily, and behavioral expressions; *The Facial Action Coding System* (FACS, Ekman and Friesen, 1978; Ekman et al., 2002), *The Maximally Discriminative Facial Movement Coding System* (MAX, Izard, 1979, 1995), *The Observed Emotion Rating Scale*^1^ (OERS, Lawton et al., 1996, 1999), *Observable Displays of Affect Scale* (ODAS, Vogelpohl and Beck, 1997), and *The Apparent Emotion Rating Instrument* (AER; Snyder et al., 1998). Two instruments employed generic approaches for emotion detection (FACS and MAX), two were dementia specific (OERS and ODAS), and one was developed to observe emotions in geriatric populations (AER).

#### Observations of Positive Expressions

Ten dementia-specific instruments that operationalized well-being as positive and negative expressions or responses to stimuli were identified; *Dementia Care Mapping* (DCM, Kitwood and Bredin, 1992), *The Positive Response Schedule* (PRS, Perrin, 1997), *Activity in Context and Time* (ACT; Wood, 2005), *Greater Cincinnati Chapter Well-Being Observational Tool* (GCC-WOT, Rentz, 2002), a revision of the former, named *Scripps Modified Greater Cincinnati Chapter Well-Being Observational Tool* (SM-GWW-WOT, Sauer et al., 2016), *AwareCare* (Clare et al., 2012), *The Behavior, Engagement and Affect Measure* (BEAM, Casey et al., 2014), *Maastricht Electronic Daily Life Observation tool* (MEDLO-tool, de Boer et al., 2016), COMMUNI-CARE (Lopez et al., 2016) and QUALIDEM-ILA (Junge et al., 2020).

#### Observations of Engagement

Seven instruments measuring engagement in dementia met the inclusion criteria; *Menorah Park Engagement Scale*^2^ (MPES, Judge et al., 2000), *Observational Measurement of Engagement* (OME, Cohen-Mansfield et al., 2009), *Music in Dementia Assessment Scales* (MiDAS, McDermott et al., 2015), *Video coding – Incorporating Observed Emotion* (VC-IOE, Jones et al., 2015), *Engagement of a Person with Dementia Scale* (EPWDS, Jones et al., 2018), *Ethographic and Laban-Inspired Coding System of Engagement* (ELICSE, Perugia et al., 2018b), and *Music Therapy Engagement Scale for Dementia* (MTED, Tan et al., 2019).

### Evaluating Measurement Properties

Extracted data on measurement properties and study characteristics are reported in Supplementary Table 2 together with the *study specific* RoB-ratings. As most publications use data from repeated observations of the same subjects, both number of participants and number of observations are reported when available. Measurement properties are presented under three headings: (a) content validity, (b) construct validity, including structural validity, measurement invariance and hypothesis testing (for construct validity), and (c) reliability, including internal consistency, inter-rater, intra-rater or test–retest reliability, and measurement error. None of the included publications reported cross-cultural validity and responsiveness, using the methodological definition and criteria of COSMIN (see Tables 1, 2).

The ratings against quality criteria for the available evidence of the measurement properties on *instrument level* are presented in Table 4. Ten of 22 instruments had only one publication describing the development and measurement properties. More than half of the instruments were developed or tested in small samples [11 of 36 studies have *n* < 20, mean *n* = 89.4 (*SD* = 102)]. The trustworthiness of the summarized result per property evaluated by the GRADE approach (GRADE Handbook, 2013; Prinsen et al., 2018) are presented in Table 4.

**TABLE 4:** Rating against quality criteria and GRADE.

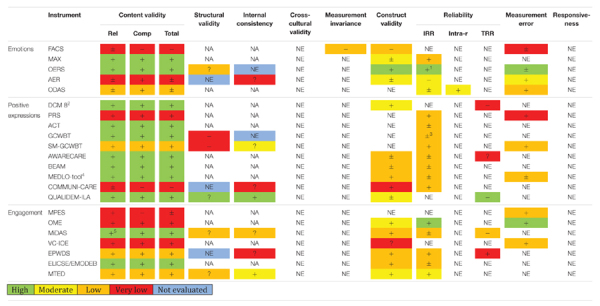

*Ratings: +, good; ?, unclear; −, inadequate; ±, conflicting; NE, not evaluated; NA, not applicable.*

*Abbreviations: Rel, relevance; Comp, comprehensiveness; IRR, Intra-rater reliability; Intra-r, Intra-rater reliability; TRR, Test–retest reliability.*

*^1^OERS: for adequately trained independent observers, IRR is good.*

*^2^DCM 8– only data regarding the well-being subscale is evaluated, and the 8th version. However, evidence of former DCM versions covers problems with inter-rater reliability (Sloane et al., 2007) and measurement invariance with dependency (Brooker, 2005).*

*^3^IRR with extensive training met criteria (Kinney and Rentz, 2005) while shorter training did not (Gross et al., 2015).*

*^4^MEDLO-tool – only the well-being/agitation subscales are evaluated. ^5^MiDAS is rated based on different timeframes in the staff (“today”) versus the music-therapist ratings (“5 min”), and it is the “momentary” ratings we focus on in this review.*

#### Content Validity

Seventeen of 22 instruments were rated as “good” when evaluated against quality criteria (MAX, OERS, PRS, DCM, ACT, GCWBT, SM-GCWBT, AwareCare, BEAM, MEDLO-tool, QUALIDEM-ILA, OME, MiDAS, VC-IOE, EPWDS, ELICSE/EMODEB, and MTED). Three instruments were rated as “conflicting” (ODAS, AER, and MPES), and two were rated as “inadequate” (FACS and COMMUNI-CARE). The study specific methodological approach for establishing content validity is presented in Supplementary Table 2.

As presented in Table 4,11 of the 17 instruments meeting quality criteria, were supported with high quality ratings of evidence of content validity according to GRADE (MAX, OERS, DCM 8, ACT, GCWBT, AwareCare, BEAM, MEDLO-tool, QUALIDEM-ILA, MiDAS, and ELICSE/EMODEB). Inviting people with dementia and/or family caregivers to include their view on the content of the instruments is an advantage, but was only conducted for AwareCare, QUALIDEM-ILA, MiDAS, and EPWDS.

#### Structural Validity and Internal Consistency

Statistical methods to investigate structural validity are only developed for unidimensional and reflective instruments or subscales and require independent observations and large samples (de Vet et al., 2011). We identified nine scales as reflective (OERS, AER, GCWBT, SM-GCWBT, COMMUNI-CARE, QUALIDEM ILA, EPWDS, and MTED). Six of the nine scales used factor analysis to investigate structural validity. Except for OERS (Lawton et al., 1996) and QUALIDEM-ILA (Junge et al., 2020), all scales are at risk of bias due to small samples (<100, GCWBT; Gross et al., 2015; SM-GWWBT; Lokon et al., 2019; MiDAS; McDermott et al., 2014; MTED; Tan et al., 2019). Use of repeated (dependent) observations of the same individuals violates statistical assumptions of these methods as well (MiDAS; McDermott et al., 2014). As Table 4 shows, no instruments have higher than “unclear”-rating of structural validity. This is mainly due to a lack of reporting model fit (OERS, QUALIDEM-ILA, MiDAS, and MTED). Investigations of structural validity for GCWBT (Gross et al., 2015) and SM-GCWBT (Lokon et al., 2019) did not confirm the theoretical factor structure.

Internal consistency was sometimes reported when no evidence of unidimensionality was provided (EPWDS; Jones et al., 2018; COMMUNI-CARE; Lopez et al., 2016; AER; Snyder et al., 1998). These results are rated as “unclear,” as internal consistency is a reliability parameter relevant for reflective instruments known to be unidimensional only (Prinsen et al., 2018).

#### Cross-Cultural Validity

No instruments reported cross-cultural validity. Nevertheless, instruments were developed in several different countries (see Supplementary Table 2), and eight reported the ethnicity of the included participants (BEAM; Casey et al., 2014; AwareCare; Clare et al., 2012; OME; Cohen-Mansfield et al., 2009; GCWBT; Kinney and Rentz, 2005; MAX; Magai et al., 2002; MiDAS; McDermott et al., 2014; MTED; Tan et al., 2019; ACT; Wood et al., 2005).

#### Measurement Invariance

Little evidence of measurement invariance was reported, when using COSMIN criteria. Only multiple group factor analysis and regression analysis are applicable approaches (Prinsen et al., 2018). An exception was FACS, where apathy explained lower frequency of facial emotions in people with mild to moderate dementia (Seidl et al., 2012).

Measurement invariance has important implications for interpretations of the scores of an instrument. Typical relevant covariates investigated were dementia severity, assessed with Pearson’s or Spearman’s correlations. Due to the methodological approach employed, these are reported under “construct validity” (Cfr. Supplementary Table 2). Lower well-being was correlated with dementia severity in MAX (Magai et al., 1997), AER (Snyder et al., 1998) and AwareCare (Clare et al., 2012). Evidence from earlier DCM-versions have shown well-being scores to vary due to level of cognitive impairment or dependency in the observed persons (Brooker and Surr, 2006; Chaudhury et al., 2013). QUALIDEM-ILA (Junge et al., 2020) and MTED (Tan et al., 2019) did not vary with dementia severity. Apathy correlated negatively with engagement in EPWDS (Jones et al., 2018).

#### Hypothesis Testing for Construct Validity

Sixteen of 22 instruments investigated construct validity through hypothesis testing. Nine instruments were thus rated as “good” (> 75% of hypotheses supported; OERS, DCM 8, MEDLO-tool, COMMUNI-CARE, OME, MiDAS, EPWDS, ELICSE/EMODEB, and MTED). Five instruments were rated as “conflicting” (MAX, AER, AwareCare, BEAM, and QUALIDEM-ILA), and one as “inadequate” (FACS). Only OERS provided evidence rated as high quality according to GRADE.

A frequently detected risk of bias was lack of specific hypotheses about the strengths of correlations with similar or divergent measures, postulated *a priori* (Prinsen et al., 2018). According to our quality criteria, significant correlations ≤ 0.3 were discarded. Weak statistically significant correlations with instruments measuring similar constructs are not adequate evidence of construct validity (Mokkink et al., 2017), but were reported as evidence supporting construct validity in AER, BEAM, and QUALIDEM-ILA.

In three of the instruments with “conflicting” evidence (AwareCare, BEAM and QUALIDEM-ILA), proxy-reported long-term QoL ratings by staff and/or family members and momentary observations by independent observers did not correlate and consequently did not support construct validity (Clare et al., 2012; Casey et al., 2014; Junge et al., 2020). Overall, further investigation of construct validity with specific and *a priori* hypotheses is required for all instruments, except OERS.

#### Inter-Rater Reliability and Measurement Agreement

As Table 4 demonstrates, some evidence of agreement between coders were reported in all but DCM 8 and QUALIDEM-ILA. Eight of 22 instruments (MAX, OERS, PRS, SM-GCWBT, COMMUNI-CARE, OME, EPWDS, and MTED) met quality criteria of inter-rater reliability (IRR, > 0.70). Of these, only two (OERS and OME) were evaluated with high quality evidence according to GRADE. Some report IRR using invalid methods according to Prinsen et al. (2018) such as Spearman’s Rho (BEAM; Casey et al., 2014) or Pearson’s correlations (GCWOT; Gross et al., 2015). For instruments concerned about item levels, the items’ specific Kappa values are the relevant parameters (Prinsen et al., 2018), but some report Kappa values on instrument level rather than an item-specific Kappa (GCWBT; Kinney and Rentz, 2005; COMMUNI-CARE; Lopez et al., 2016; SM-GCWBT; Sauer et al., 2016; PRS; Schall et al., 2015).

If the total sum of the scale is to be used, IRR should be assessed with intra class correlations (ICC), as the agreement of the *total sum* is the relevant reliability parameter (Prinsen et al., 2018). For most health measurement instruments, the preferred ICC formula is absolute agreement for random models with single measurements. This reflects whether different observers consistently reach the same conclusions (see de Vet et al., 2011; chapter 5). However, the formulae were often not reported and suboptimal calculations were often used.

For ordinal, nominal and dichotomous level scores, measurement error is defined as measurement agreement between raters. This was reported for 10 instruments, where seven met the quality criteria (> 80%, AER, ODAS, PRS, SM-GCWBT, MPES, OME, and VC-IOE). Of these, only one was evaluated with high quality evidence (OME).

Low inter-rater agreement (IRR and measurement agreement) may reflect both lack of training and problems with content validity/poor operationalizations of the items. The amount of training will affect the level of inter-rater agreement, for instance as shown in OERS (Lawton et al., 1999) and when comparing inter-rater reliability for GCWBT with extensive training (Kinney and Rentz, 2005) and 30 min training (Gross et al., 2015). For MiDAS, the varying timeframes of the staff- and music-therapist ratings (“today” versus 5 min) may account for the low inter-rater reliability of the staff-ratings (McDermott et al., 2014). This may well reflect lower relevance of the items in the prolonged timeframe, and potentially issues concerning content validity.

#### Test–Retest Reliability and Measurement Error

Test–retest reliability was rarely investigated, and of the five scales reporting on this property, EPWDS was the only scale meeting the quality criteria. To validly evaluate test–retest reliability, the subjects need to be stable in the interim-period to ensure that any difference is caused by random measurement error (de Vet et al., 2011). In general, several studies showed fluctuating well-being scores (AwareCare; Clare et al., 2012; QUALIDEM-ILA; Junge et al., 2020; MiDAS; McDermott et al., 2014). Competing explanations of low test–retest reliability may include too long an interval between comparison measurements or may simply reflect qualities of the construct.

The low test–retest reliability detected for DCM 8 is prone to bias, as the assessments were three months apart (Villar et al., 2015).

For continuous level scores, measurement error is related to the test–retest reliability, and we need to know the smallest detectable change (SDC) or limits of agreement (LoA), as well as the minimal important change (MIC) defined by the target group, to apply the quality criteria (Prinsen et al., 2018). None of the instruments reported these outcomes.

#### Responsiveness

No instruments reported evidence of responsiveness.

### Feasibility and Interpretability

Extracted data regarding feasibility and interpretability are reported in Supplementary Table 3. Additional publications from the search process describing use of the instrument in clinical settings or research were extracted here.

#### Feasibility

Four instruments require video-recordings (FACS, ODAS, VC-IOE, and ELICSE) and the latter may be used for direct observation. Several instruments allow for observing people simultaneously or sequentially (DCM, ACT, GCWBT, SM-GCWBT, BEAM, MEDLO-tool, MiDAS, and MTED). Some instruments were developed mainly as research tools (FACS, MAX, ODAS, PRS, ACT, VC-IOE, and ELICSE). Two instruments appear best suited for evaluation in care settings only (DCM 8 and MTED). Several instruments appear feasible for evaluating psychosocial interventions (FACS, MAX, ODAS, OERS, AER, PRS, ACT, MPES, BEAM, and QUALIDEM-ILA), and some are suited for care settings as well (OERS, AER, ACT, BEAM, QUALIDEM-ILA, DCM 8, AwareCare and MEDLO-tool). Some instruments are developed for *specific* interventional approaches, including art-interventions and other creative interventions (GCWBT and SM-GCWBT), multi-sensory interventions (COMMUNI-CARE), interaction with social robots (ELICSE, VC-IOE, and EPWDS), and music interventions (MiDAS and MTED). Most instruments are feasible for persons with mild, moderate, and severe dementia, but two instruments were specifically developed for very severe dementia (PRS and AwareCare). Personalized stimuli can be incorporated in two instruments (AwareCare and OME), and six instruments are easily adapted to other environmental contexts (OERS, GCWBT, SM-GCWBT, BEAM, MEDLO-tool, and ACT).

#### Interpretability

Skewed distributions of the negative expressions were commonly reported (FACS/EMFACS; Asplund et al., 1995; ODAS; Beck et al., 2002; Beerens et al., 2016; BEAM; Casey et al., 2014; MEDLO-tool; de Boer et al., 2016; MPES; Judge et al., 2000; GCWOT; Kinney and Rentz, 2005; OERS; Lawton et al., 1999; SM-GCWOT; Lokon et al., 2019; MAX; Magai et al., 1996, 2002; PRS; Perrin, 1997; Phillips et al., 2010; ACT; Wood, 2005). For AwareCare, infrequent items were removed during fieldwork to avoid skewness (Clare et al., 2012).

Sensitivity to detect statistically significant changes were demonstrated for FACS/EMFACS (in people with mild to moderate dementia; Seidl et al., 2012; but not for people with severe dementia; Asplund et al., 1995), MAX (Magai et al., 1996), OERS (when aggregating positive and negative affect; Hammar et al., 2011; except anger; Lawton et al., 1999), AER (Snyder et al., 2001), ODAS (for two of three subscales, Beck et al., 2002, or when aggregating scores to positive and negative affect; Lee et al., 2013, 2014, 2017), DCM 8 (Brooker, 2005), PRS (Hadley et al., 1999; Schall et al., 2015), ACT (Wood et al., 2005; Lassell et al., 2021), GCWBT (positive items only, Kinney and Rentz, 2005) and SC-GWBT (Sauer et al., 2016; Lokon et al., 2019), AwareCare (Clare et al., 2012, 2014), BEAM (for “happiness” and “agitation”, Low et al., 2014), MEDLO-tool (“mood”; Beerens et al., 2016, 2018), MPES (Lee et al., 2007), OME (Cohen-Mansfield et al., 2011, 2012), MiDAS (Garrido et al., 2020) and EPWDS (Feng et al., 2020).

To ease interpretation, available sources for means and standard deviations of scores are reported in Supplementary Table 3. However, guidelines for interpretation of *clinically significant* scores or change scores are not identified in most instruments. DCM 8 offers calculating an individual or group level well-being profile. PRS gives a ratio, where higher ratios imply the setting triggers more well-being. AwareCare offers calculation of a “Responsiveness Index” for stimuli or for the individual, enabling the assessment of both individual processes and comparisons on group-level (Clare et al., 2012). COMMUNI-CARE provides a cut-off score of positive, indifferent, and negative effects of an intervention (Lopez et al., 2016). For ACT and EPWDS, creating an individual baseline is recommended to interpret change-scores.

## Discussion

In this review we investigated observational instruments assessing momentary well-being in the context of research, interventions and care for people living with dementia. We identified 22 instruments, and evaluated RoB on study level, and measurement properties, feasibility, and interpretability on instrument level. The content validity of many of the instruments reviewed was sound and supported by high quality evidence for 11 instruments. Meanwhile, the presence of high-quality evidence of other central psychometric aspects was sparse. This may in part be explained historically by the more recent development of stringent quality criteria. Hence, several instruments have the potential to meet these quality criteria if further investigated. To guide and advise further use of these instruments in care and research, we provide a general discussion of the most common methodological problems. Finally, we present instrument-specific recommendations.

### Issues Regarding Measurement Properties, Feasibility, and Interpretability

Problems with skewed distributions or low frequencies of negative emotions, behaviors or expressions are reported for the majority of the instruments (Cfr. Supplementary Table 3). This complicates parametric approaches assuming a normal distribution of items. We suggest that assessing psychosocial interventions for people living with dementia should mainly focus on *increases* in well-being. Negative symptoms in dementia have a diversity of causes, some of which will necessarily be less modifiable by psychosocial interventions (Kales et al., 2015; Kolanowski et al., 2017; Livingston et al., 2017). However, momentary well-being is particularly achievable through modifying environmental factors (Lawton, 1994; Kolanowski et al., 2020). Moving the focus from ill-being (such as agitation or apathy) to well-being, has three advantages. First, it will decrease the labor intensiveness of the observational assessment because less items are assessed. Second, it will bring about data better fitted for statistical approaches because the distribution of ill-being items in the clinical studies using these instruments often were skewed and not normally distributed (see Asplund et al., 1995; Magai et al., 1996, 2002; Perrin, 1997; Lawton et al., 1999; Judge et al., 2000; Beck et al., 2002; Kinney and Rentz, 2005; Wood, 2005; Phillips et al., 2010; Casey et al., 2014; Beerens et al., 2016; de Boer et al., 2016; Lokon et al., 2019). Lastly, it will increase the likelihood of correct conclusions about the positive effects of the psychosocial interventions because this is operationally defined as an increase in positive expressions and not as a decrease in negative expressions. Ill-being should still be monitored during psychosocial interventions, but the absence of ill-being is not synonymous with well-being (Martyr et al., 2018).

While 15 of 22 instruments could detect statistically significant changes, definitions to guide *interpretation* of these change-scores were not provided. An option for future studies is to calculate MIC and the SDC or LoA (de Vet et al., 2011) for continuous level instruments. MIC is important because it is defined as the smallest clinical meaningful change as evaluated by patients or clinicians (de Vet and Terwee, 2010). SDC indicates whether change scores are reflecting a “true” change in the construct, as opposed to expected random error or natural fluctuation. Test–retest values may be used to calculate SDC for continuous scores (Prinsen et al., 2018; Mokkink et al., 2020). Several instruments were operationalized at a nominal or ordinal level, while using total score as continuous in statistical analyses. However, using the total score implies that the score reflects, predicts, or describes well-being validly. Although several instruments claim the total score to reflect level of well-being or engagement, adequate evidence of this relationship is rarely provided. Specifically, the formative instruments are hampered by unclear clinical interpretation.

Test–retest reliability reflects the instrument’s measurement error in repeated measurement of stable constructs (de Vet et al., 2011). This required “stability” may be unattainable for fluctuating phenomena such as pain. In this review, several instruments provide evidence suggesting momentary well-being in dementia is a fluctuating phenomenon (Clare et al., 2012; McDermott et al., 2014; Junge et al., 2020). Fluctuations in the construct of interest between measurements creates an ambiguous reliability estimate (Jensen, 2003) and discarding instruments with a cut-off score < 0.70 (Prinsen et al., 2018) is not necessarily useful in this context. It is reasonable to assume test–retest scores reflect a natural fluctuation or variability in well-being in people with dementia, as the presence of neuropsychiatric symptoms such as apathy are episodic and fluctuating as well (Kales et al., 2015). Examining the natural variation of the construct by investigating test–retest reliability is nevertheless important, as the range of variation in fluctuating constructs influence the accuracy when interpreting scores of an instrument. Thus, a clinically significant score needs to be larger than the measurement error inflicted by this natural variation (de Vet et al., 2011). If test–retest reliability is not investigated, we cannot know if the measure can detect change in the observed persons beyond measurement error (Mokkink et al., 2020). This is a significant problem, that may lead to erroneous conclusions in both research and care. In addition, adjusting the interval of the repeated measurements to increase the likelihood of stability is essential, as longer time intervals may reflect the degenerative path of dementia and not instrument reliability.

Developing fine grained instruments used for ecological momentary sampling requires repeated assessment of the same subjects (Shiffman et al., 2008). Investigating behavior as it unfolds over time is labor intensive, and naturally includes smaller samples, often with numerous repeated observations. Standard approaches to develop self-rated instruments require large samples to investigate structural validity with factor analysis (*N* > 100), or scalability through for example Mokken analysis (*N* > 2000; Prinsen et al., 2018). Investigating large samples in labor intensive instruments is in many cases unrealistic. Additionally, using serially dependent repeated observations in the same subjects to increase the sample size violates basic assumptions required for these methods (Manolov and Moeyaert, 2017).

Most instruments in this review require further investigations of construct validity to ensure that the output is consistent with the underlying theoretical constructs. Comparisons with global rating scales are recommended when investigating the construct-validity of new instruments (de Vet et al., 2011). While developing COMMUNI-CARE, a validated clinician-rated global scale was used for this purpose (Lopez et al., 2016), but the same non-blinded investigator was rating both scales, contributing to a considerable risk of bias. In OME (Cohen-Mansfield et al., 2009), a similar approach is used, only with blinded ratings of a non-validated global engagement-scale. Thus, investigating construct validity through correlations with similar instruments is a challenge in the face of a lack of a “gold standard measure,” as one must rely on existing instruments with their respective limitations (de Vet et al., 2011). Sometimes the hypothesized correlations included comparisons of well-being levels from long-term versus momentary instruments (Clare et al., 2012). Well-being states and traits do not necessary correlate (Curyto et al., 2008; Cohen-Mansfield, 2011). Therefore, investigating correlations with other momentary assessment approaches is recommended.

When assessing momentary well-being in dementia, two domains seem important to control for to interpret changes in well-being scores more accurately. Several of the instruments included in this review have a well-being score that is associated with (1) dementia severity or (2) level of function. However, research suggests that these constructs are not expected to be systematically related (Missotten et al., 2008; Barca et al., 2011; Cohen-Mansfield, 2011; Martyr et al., 2018). This has implications for how we interpret changes in well-being scores over time. If well-being scores of a particular instrument are lowered as a consequence of the dementia progressing, is this reflecting lack of treatment effect, poorer dementia care, or neurodegenerative development? Future studies assessing the measurement properties of these instruments should assess if a relationship between well-being and dementia severity or level of function is present. Such covariance may indicate that the instrument is tapping both cognitive functioning as well as well-being (for example if the score is relying on verbal expression). Understanding these relationships is required to accurately interpret changes in well-being scores during psychosocial interventions.

Personal well-being refers to a subjective evaluation, and observational measures use behavioral expressions to infer about an inner state. Hence, the most crucial property of a measurement instrument is *content validity*. Content validity will vary with the context, population, and construct to be measured, and affects all other psychometric properties of an instrument (Terwee et al., 2018). Together with agreement between observers, these two aspects are considered the most important for observational instruments (Bakeman and Quera, 2012; Chorney et al., 2015). Moreover, evidence of structural validity or construct validity, ensuring that an increase in the score reflects an increase in the construct, is important when making inferences about inner states. Cross-validating scores with other instruments, particularly self-report instruments, will strengthen this.

As no evidence of cross-cultural validity or responsiveness was detected, special attention to investigating this knowledge-gap and establishing these properties are important in future studies using any of the instruments in this review. In relation to cross-cultural validity, we make the following recommendation: Behavioral expressions of momentary well-being are likely to differ across cultures (Lim, 2016). Thus, securing cross cultural validity by *establishing content validity in new cultural contexts* is in our evaluation an alternative to statistical evaluation of cross-cultural validity for observational measures. This can be achieved through the recommended qualitative approaches involving clinical expertise from people with dementia, family- and professional caregivers, as well as clinical experts and field testing (Terwee et al., 2018).

In relation to the lack of responsiveness, we make the following recommendation: Several instruments have provided evidence of their capacity to statistically detect changes in intervention studies (Conf. Supplementary Table 3). However, this is not adequate evidence of responsiveness, as we do not know if the lack of detecting change is due to lack of responsiveness or lack of intervention effect. Responsiveness of these instruments needs to be investigated through correlations with change-scores in similar instruments (de Vet et al., 2011).

The *clinical utility* of an instrument is specific to the context and aims of the user, and is influenced by its feasibility, interpretability, benefits, and shortcomings (Smart, 2006; Terwee et al., 2018). To recommend a specific instrument to assess observed well-being is not our intention. However, we generally recommend identifying instruments with proper conceptualizations, which are feasible for the specific purpose, context, and target population (Terwee et al., 2018). Choosing instruments with acceptable content validity should be followed by investigation or adaptation to solve the additional instrument-specific issues addressed in this review. An overview of the issues of each instrument is provided in Table 4, Supplementary Tables 2, 3. Establishing or evaluating if the instrument has good content validity in the applied context is vital, especially in securing relevance and comprehensiveness (Chorney et al., 2015).

A final note worth commenting regards the large number of instruments identified in the hand search, of which three were included in this review. This suggests that researchers may not be choosing appropriate keywords when publishing articles relating to observational measures for people living with dementia.

### Recommendations of Instruments

Of the instruments measuring *emotions* with acceptable content validity (OERS and MAX), OERS is the most frequently used (Lee et al., 2019) instrument with the most extensively documented psychometric properties (Lawton et al., 1996, 1999). MAX (and FACS) requiring a close view of the face; problems with interpreting facial movement in persons wearing glasses, having facial hair, or facing more than 45 degrees away from the camera (Cohn et al., 2007) reduces the clinical utility of these instruments in people living with dementia. Thus, the feasibility of instruments relying on facial expressions and excluding bodily expressions may decrease the instruments’ sensitivity to detect expressions of well-being in the dementia population (Seidl et al., 2012). However, as negative emotions are infrequent, the feasibility of the full OERS scale in research and clinical setting is limited (Algar et al., 2016). Thus, for investigating well-being in people with dementia, the positive emotions in OERS may be best suited. However, from these findings, emotions in people with mild to moderate dementia seem to be best measured through self-report (instruments are reviewed in Ferring and Boll, 2010; Stoner et al., 2019; and Clarke et al., 2020).

Users looking for instruments investigating *positive expressions* are recommended to consider any instruments with acceptable content validity (DCM 8, PRS, ACT, GCWBT, SM-GCWBT, AwareCare, BEAM, MEDLO-tool and QUALIDEM ILA). PRS and MEDLO-tool are instruments with high granularity, detecting changes on micro-levels that offer interval-sampling from 30 s to 2 min. While DCM, ACT, GCWBT, SM-GCWBT offers somewhat fine-grained observations (5-10 min), AwareCare offers fine-grained observations as they unfold over time, and BEAM consists of both fine-grained and aggregated scores. QUALIDEM-ILA is best suited for total evaluations of interventions (30-45 min). Users looking for behavioral or movement-anchored operationalizations of positive expressions with high levels of concreteness may look at PRS, ACT and AwareCare. DCM, GCWBT, SM-GCWBT, BEAM, MEDLO-tool and QUALIDEM-ILA offer more contextual cues and social interpretations.

AwareCare appears clinically useful for people with very severe dementia, and BEAM is feasible for moderate dementia. AwareCare detected signs of awareness in all participants and suggests a clinically useful index for interpretation as well (Clare et al., 2012). PRS needs to be investigated in a larger sample but is a promising tool in very severe dementia (Perrin, 1997). BEAM covers behavior, engagement, and affect, through direct observation in various settings without being very labor intensive and while avoiding observer’s fatigue (Casey et al., 2014). Further investigation of its construct validity may, however, be required, in addition to an improved evaluation of inter-rater reliability. The clinical sensitivity of DCM has been questioned (Cooke and Chaudhury, 2013), and the well-being (ME-score) of DCM 8 is probably not sensitive enough to detect clinical change reliably in intervention studies on a group level. DCM 8 seems better suited for clinical practice (Villar et al., 2015) on an individual level (Brooker and Surr, 2006). MEDLO-tool’s mood score is based on DCM as well, and shows the same problems (Beerens et al., 2016; de Boer et al., 2016), lowering the utility of this instrument for assessing well-being. ACT is based on a thorough development (Wood, 2005), and seems like a feasible and clinically useful instrument, but needs further investigation of construct validity. GCWBT should be omitted due to evidence of low structural validity (Gross et al., 2015), but the revised SM-GCWBT needs further modification and investigation of a proposed two-factor structure, as well as exclusion of some unrelated items (Lokon et al., 2019). Further investigation of QUALIDEM-ILA, in terms of both inter-rater reliability and use in clinical/research contexts are required (Junge et al., 2020). Still, QUALIDEM-ILA is one of the most recent instruments included in this review, and further publications are expected.

Of the instruments assessing *engagement* with acceptable content validity (OME, VC-IOE, EPWDS, ELICSE, MiDAS, and MTED), users searching for instruments with high granularity may look at VC-IOE or ELICSE (both continuous sampling), EPWDS or MiDAS (5-min intervals), or OME (15 min including both duration-based and aggregated scores). MTED provides an aggregated score based on the intervention-session. ELICSE and VC-IOE offers the highest level of concreteness, and EPWDS, OME, MiDAS, and MTED is less concrete and more interpretative. However, higher levels of concreteness will often increase labor intensiveness (Bakeman and Quera, 2011) and offer broader generalizability, at the cost of lower sensitivity to individual variations. In clinical contexts, allowing for interpreting idiographic expressions of well-being may sometimes be an advantage.

Ethographic and Laban Inspired Coding System of Engagement (ELICSE) is based on an exemplary solid development-phase with subsequent theoretical and conceptual development (Perugia et al., 2018a,b, 2020). Nevertheless, the system is highly context specific to the manipulation of objects when sitting down and may not be as easily adaptable to other activities or clinical contexts. Developers of OME describe a need for further work on increasing the clinical utility of the scale (Cohen-Mansfield et al., 2011, 2012), and it is critiqued for lack of interpretability (Jones et al., 2015, 2018; Perugia et al., 2018b). VC-IOE needs further evaluation of reliability and construct validity (Jones et al., 2015). MiDAS strength is the inclusion of the target group in the development (McDermott et al., 2015), but needs further investigation of psychometric properties and is hampered by low intra-rater reliability (McDermott et al., 2014). MTED appear to be a good option when evaluating engagement in clinical music therapy processes, but the scale is not intended for evaluating intervention effect (Tan et al., 2019).

Engagement of a Person with Dementia Scale (EPWDS) stands out as a feasible, easily administered scale that may allow for assessing engagement in contexts other than robot-based play (Jones et al., 2018). Formal evaluation of its structural validity is required, but indications of test–retest reliability are promising given the common problems of low stability between assessments in this population.

### Strengths and Limitations

The first strength of this review is that the protocol was pre-registered in PROSPERO. The second strength is that we used the most relevant systematic approach, the COSMIN-guidelines (Prinsen et al., 2018; Terwee et al., 2018; Mokkink et al., 2020). The third strength is that when required, these guidelines were adapted for evaluating observational instruments based on relevant literature (Bakeman and Quera, 2011, 2012; Chorney et al., 2015; Perugia et al., 2018b). The fourth strength is the extensive review of study-specific and instrument-specific evaluation and overarching methodological issues that provides relevant knowledge to both researchers and practitioners.

A first limitation of this review is that by including instruments reporting at least one psychometric property, instruments describing promising content validity only were not evaluated (such as Morse and Chatterjee, 2018). A second limitation is that the COSMIN-criteria of construct validity requires at least 75% of hypotheses to be supported. This may lead to somewhat unbalanced ratings, as publications reporting only one or two supportive correlations may be given a more positive rating than studies examining multiple correlations. However, testing several hypotheses provides more detailed knowledge about construct validity. A third limitation is the use of correlations of > 0.3 as the cutoff for supporting construct validity. This cutoff may seem low, and less conservative than the original suggestion of correlations ≥ 0.5 with instruments measuring similar constructs (Prinsen et al., 2018). However, the majority of the correlated instruments were assessing related and not similar constructs, indicating that correlations > 0.3 are an adequate expectation. Finally, the blinding procedure within our team of raters could have been more extensive, as completely blinded ratings are considered the gold standard (Mokkink et al., 2017).

### Conclusion

Several instruments may validly assess well-being through observation in people with dementia. Evaluating their context specific clinical utility and content validity are more important than choosing the instrument with the best ratings or psychometric properties. However, piloting the instruments, investigating the effects of cultural context and study-specific inter-rater agreement and measurement error is advised. Moreover, utilizing an instrument in a clinical study provides the opportunity to investigate hypotheses that may further inform the construct validity. All measurement approaches come with some strengths and some weaknesses, and observational measures are vulnerable to misinterpretation when they are used to infer about inner states. Nevertheless, observations offer unique opportunities to investigate associations between external stimuli and well-being that can provide important knowledge of the usefulness of various interventions for people living with dementia.

## Data Availability Statement

The original contributions presented in the study are included in the article/Supplementary Material, further inquiries can be directed to the corresponding author/s.

## Author Contributions

KM conducted the literature searches, initial screening of records and imported these to the data-management-tools, extracted data, assessed RoB, evaluated against quality criteria, and conducted GRADE-ratings, and wrote methods and result-section, with ideas and commentaries from EF-G and IN. IN and KM blind-screened the abstracts for inclusion and adapted the COSMIN guidelines to observational measures. These were consecutively reviewed in consensus-meetings with KM and IN. EF-G were included in discussions if consensus was not met. KM, EF-G, and IN reviewed and consolidated extracted data into the current tables and wrote the introduction and discussion in collaboration. NP edited the document for conceptual clarity and discussed methodological and quantitative considerations regarding the measures. All authors contributed to the article and approved the submitted version.

## Conflict of Interest

The authors declare that the research was conducted in the absence of any commercial or financial relationships that could be construed as a potential conflict of interest.

## Publisher’s Note

All claims expressed in this article are solely those of the authors and do not necessarily represent those of their affiliated organizations, or those of the publisher, the editors and the reviewers. Any product that may be evaluated in this article, or claim that may be made by its manufacturer, is not guaranteed or endorsed by the publisher.

## Footnotes

1 Variations of the name of the OERS are Philadelphia Geriatric Center Affect Scales, Apparent Affect Rating Scale, Lawton’s Modified Behavior Stream, Affect Rating Scale, and Observed Affect Scale (Lee et al., 2019).

2 MPES is also referred to as Myers Research Institute Engagement Scale (Lee et al., 2007).
