# Supplementary material for: Assessing Momentary Well-Being in People Living With Dementia: A Systematic Review of Observational Instruments
Source: Front Psychol. 2021 Nov 23;12:742510. doi: 10.3389/fpsyg.2021.742510 (PMC8649635; doi:10.3389/fpsyg.2021.742510)
Supplement: Supplementary file 1 [file Data_Sheet_1.docx]

Supplementary Material

# Supplementary Data

**Table S1** *Adapted RoB-ratings for COSMIN Box 1, 2 and 3*

|  | Adapted Cosmin guidelines for evaluating the content validity of Observer-Reported Outcome Measurement instruments (ObsROMs)^[[1]](#footnote-1)^ |
| --- | --- |
|  | Box 1 – ObsROM development |
| *1a* | *Instrument design* |
| *1* | *Is a clear description provided of the construct to be measured?* |
| *2* | *Is the origin of the construct clear: was a theory, conceptual framework or disease model used or clear rationale provided to define the construct to be measured?* |
| *3* | *Is a clear description provided of the target population for which the* ObsROM *was developed?* |
| *4* | *Is a clear description provided of the context of use (i.e., discriminative, evaluative purpose, and/or predictive)* |
| *5* | *Was the* ObsROM *development study performed in a sample representing the target population for which the instrument was developed?* |
|  | *Concept elicitation* |
| *6* | *Was an appropriate* qualitative or quantitative *data collection method used to identify relevant* items *for a new* ObsROM^[[2]](#footnote-2)^? |
| *7* | If qualitative: *Were skilled group moderators/interviewers/* observers/ raters *used?* |
| *8* | If qualitative interviews: *Were the group meetings or interviews based on an appropriate topic or interview guide, interviews were recorded and transcribed verbatim?* |
| *9* | If qualitative: *Was at least part of the data coded independently? (11-49% = A)* |
| *10* | If qualitative: *Was data collection/fieldwork continued until saturation was reached?* |
| *11* | If theoretical approach: Was a literature review or conceptual framework clearly described and a thorough representation of the research field? |
| *12* | *For quantitative studies (i.e., survey or Delphi procedure): Was the data collection continued until consensus was reached?* |
| *13* | *For quantitative studies* (i.e., survey or Delphi procedure): *was the sample size appropriate?* |
| *14* | If qualitative fieldwork: Was a methodological sound approach to develop coding scheme or ethogram applied |
| *15* | Were at least two appropriate approaches to developing the instrument used (qualitative fieldwork using observation, qualitative interviews/focus groups, theoretical approach building on former instruments or theoretical development, OR quantitative survey/Delphi approach with relevant experts (patients, family-caregivers, or clinicians) |
| *16* | *Was an appropriate* and transparent *approach used to analyze the data?* |
| *1b* | *Pilot test/development phase* |
| *17* | *Was pilot test performed?* |
|  | *General design requirements* |
| *18* | *Was* pilot testing *performed in a sample representing the target population* and study context*?* |
| *19* | Were operational definitions refined based on pilot-testing? |
| *20* | *Was each item tested in an appropriate number of patients? For qualitative studies ≥7*  *For quantitative (survey) studies ≥50* |
|  | *Comprehensibility* |
| *21* | *Were problems regarding the comprehensibility of the instrument instructions, items, response options, and recall period appropriately addressed by adapting the instrument?* |
| *22* | *Were all the items tested in their final form?* |
|  | *Box 2. Content validity^[[3]](#footnote-3)^* |
|  | *Asking patients* or family caregivers *about relevance^[[4]](#footnote-4)^* |
| *1* | *Was an appropriate method used to ask patient or* family caregivers *whether each item is relevant for their experience with the condition?* |
| *2* | *Was each item tested in an appropriate number of patients? For qualitative studies ≥7*  *For quantitative (survey) studies ≥50* |
| *3* | If qualitative***:*** *Were skilled group moderators/interviewers used?* |
| *4* | If qualitative interviews: *Were the group meetings or interviews based on an appropriate topic or interview guide, interviews were recorded and transcribed verbatim?* |
| *5* | *Was an appropriate approach used to analyses the data?* |
| *6* | If qualitative: *Were at least two researchers involved in the analysis?* |
| *7* | For quantitative studies (i.e., survey or Delphi procedure): Was the data collection continued until consensus about relevance was reached? |
|  | *Asking patients* or family caregivers *about comprehensiveness* |
| *8* | *Was an appropriate method used to for assessing the comprehensiveness of the* ObsROM |
| *9* | *Was each item tested in an appropriate number of patients? For qualitative studies ≥7*  *For quantitative (survey) studies ≥50* |
| *10* | If qualitative: *Were skilled group moderators/interviewers used?* |
| *11* | If qualitative interviews: *Were the group meetings or interviews based on an appropriate topic or interview guide, interviews were recorded and transcribed verbatim?* |
| *12* | *Was an appropriate approach used to analyze the data?* |
| *13* | If qualitative: *Were at least two researchers involved in the analysis?* |
| *14* | For quantitative studies (i.e. survey or Delphi procedure): Was the data collection continued until consensus about comprehensiveness was reached? |
|  | *Asking professionals about relevance,* or investigating relevance with other approach |
| *15* | Was the relevance of items secured by an appropriate method? (Fieldwork/ethogram, adapting other coding schemes, theoretical approach, literature search, qualitative interviews or quantitative surveys involving professionals?) |
| *16* | If survey or qualitative interview: *Were professionals from all relevant disciplines included?* |
| *17* | *Was each item tested in an appropriate number of professionals* or subjects*? For qualitative studies ≥7*  *For quantitative (survey) studies ≥50* |
| *18* | If qualitative approach: *Were skilled group moderators/interviewers /raters/ observers used?* |
| *19* | If qualitative interviews:  *Were the group meetings or interviews based on an appropriate topic or interview guide, interviews were recorded and transcribed verbatim?* |
| *20* | If qualitative: *Were at least two researchers involved in the analysis?* |
| *21* | *Was an appropriate approach used to analyze the data?* |
| *22* | For quantitative studies (i.e. survey or Delphi procedure): Was the data collection continued until consensus about relevance was reached? |
|  | *Asking professionals about the comprehensiveness,* or investigating comprehensiveness with other approach |
| *23* | Was an appropriate method used for assessing the comprehensiveness of the instrument (Fieldwork/ethogram, adapting other coding schemes, theoretical approach, literature search, qualitative interviews or quantitative surveys involving professionals?) |
| *24* | If survey or qualitative interview: *Were professionals from all relevant disciplines included?* |
| *25* | *Was each item tested in an appropriate number of professionals or* subjects*? For qualitative studies ≥7*  *For quantitative (survey) studies ≥50* |
| *26* | *Was an appropriate approach used to analyze the data?* |
| *27* | If qualitative: Were skilled group moderators/interviewers/raters/ observers used? |
| *28* | If qualitative interviews: Were the group meetings or interviews based on an appropriate topic or interview guide, interviews were recorded and transcribed verbatim? |
| *29* | If qualitative: *Were at least two researchers involved in the analysis?* |
| *30* | For quantitative studies (i.e. survey or Delphi procedure): Was the data collection continued until consensus about relevance was reached? |

**Table S2** *Psychometric properties and associated RoB-ratings*

| **Reference** | **Study characteristics:**  **N of participants**  **N_o_ of observations**  **Gender (% women) Population**  **Setting**  **State/country** | **Content validity** | | **Construct validity:**  **Structural validity**  **Measurement invariance**  **Hypotheses testing for construct validity** | | **Reliability:**  **Internal consistency**  **Reliability (IRR, Intra-R)**  **Measurement error** | |
| --- | --- | --- | --- | --- | --- | --- | --- |
|  |  | Approach | RoB | Results | RoB | Results | RoB |
| **EMOTIONS** | | | | | | | |
| **FACS/EMFACS^1^** | | | | | | | |
| Asplund et al., (1991) | N = 4  N_o_ = 60  100 % women  Severe Alzheimer dementia  Setting not reported  Sweden | Generic scale. Content validity in the context of mild to moderate dementia not formally evaluated. Tested in small scale in severe dementia, concluding with some sensitivity for facial movement action units, but content validity for combinations of action units to detecting emotions (EMFACS) was low | A |  |  | *Measurement error*: Average of exact agreement 76%, range 43 – 90% | A |
| Asplund et al., (1995) | N = 4  N_o_ = 4500  50 % women  Severe Alzheimer dementia  Geriatric department  Sweden | Comparing FACS/EMFACS to naturalistic judgement showed lack of comprehensiveness of the action units detected and facial cues to be ambiguous. | A | *Hypothesis testing for construct validity:* Comparison of naturalistic (qualitative) judgements of emotions and FACS showed low agreement (48 %). Only happiness and disgust were detected by both methods | D |  |  |
| Seidl et al., (2012) | N = 47  N_o_ = not reported  37% women  Moderately severe Alzheimer’s dementia  Long term care  Germany |  |  | *Measurement invariance:* Apathy and not cognitive impairment explained frequencies of facial expression of action units (*R^2^* = 0.21) | D |  |  |
|  |  |  |  | *Hypothesis testing for construct validity (comparisons between subgroups):* People with higher scores on apathy showed less total (*r* = -60) and specific (*r* = -42) facial expression of action units (Pearson’s correlation) | V |  |  |
| **MAX** | | | | | | | |
| Magai et al., (1997) | N = 82  N_o_ =  77 % women  Moderately to severe Alzheimer’s dementia  Nursing home  New York, USA | Generic scale. Field work and hypothesis-testing provides evidence of adequate comprehensibility and relevance in people with dementia. Tested whether people with dementia retained the ability to express basic emotions and demonstrates comprehensibility and relevance for interest, happiness, sadness, and anger. Fear was infrequent during observations. | V | *Hypothesis testing for construct validity:* Correlations with emotions reported by professional caregivers for interest (r = .36*) and joy (r = .56***),  and reports from family caregivers  for interest (r = .31*), anger (r = .61***), and joy (r = 41*). Sadness was not correlated, and fear was too infrequent.  *Comparison between subgroups*: interest and joy decreased in people with more severe dementia.  7 of 10 hypotheses (70% supported) | A | *IRR*: κ ≥ .80 for individual items before direct observation. Training “Interest” = .88, “joy” = 1.00, “sadness” = .86, “contempt” = 1.00, “fear” = 1.00, “disgust” = 0.90, “knit brow” = 1.00. | D |
| Magai et al., (2002) | N = 91  N_o_ =  93 % women  Moderately to severe dementia  New York, USA |  |  |  |  | *IRR*: Total scale κ = .80 (range .72-.97) pre-assessment, and .86 (range .69 – 1.0) after 6 weeks | D |
| **OERS** | | | | | | | |
| Lawton et al., (1996) | N = 253 (+ 43 for comparison)  N_o_ = >3000  Gender not reported  Moderate Alzheimer’s dementia  Nursing home  Philadelphia, USA | Literature review, items from other scales, development of coding scheme through fieldwork, including hypothesis testing in different populations | V | *Structural validity*: A two-factor structure is suggested, but exploratory (PCA, varimax rotation) factor analysis support both a one and two-factor affect-structure | A | *IRR*: κ between .76 – .89 | V |
|  |  |  |  | *Hypotheses testing for construct validity:* A priori hypotheses describing convergent, divergent and zero-correlations. Range of significant correlations were .20 – .49. 63 of 85 hypotheses (74%) were supported and above *r* = >.3.  *Comparison between subgroups:* People with dementia scored lower on positive emotions and higher on anxiety compared to people without cognitive impairment | A |  |  |
| Lawton et al., (1999) | N = 259  N_o_ = 858  Gender not reported  Mild to severe dementia  Nursing home  Philadelphia, USA | Revised items including signs more common in persons with severe dementia | V | *Hypothesis testing for construct validity (comparison between subgroups):* Testing confirmed adequate sensitivity in varying contexts (morning care, meal-time, down-time and activity). 4 of 4 hypotheses (100%) supported | A | *IRR:* ICC in enhanced training group  “pleasure” .87, “anger” .62, “anxiety” .67, “depression” .53, “interest” .85  κ_w_ = “pleasure” .66, “anger” .37, “anxiety” .40, “depression” .29, “interest” .58 | A |
|  |  |  |  |  |  | *Measurement error*: % agreement in enhanced training group: “pleasure” 84%, “anger” 90%, “anxiety” 79%, “depression” 86%, “interest”87% | A |
| **AER** | | | | | | | |
| Snyder et al., (1998) | N = 312  N_o_ = 936  71% women  Geriatric population  Nursing home  Minnesota, USA | Scale developed based on literature review and building on former scale(s). Scoring of items has low face validity, and no pilot test for relevance or comprehensiveness is reported | I | *Hypothesis testing for construct validity:* Pearson’s correlation with depression *r*= -.19** and affect *r* = .30**. More cognitive impaired persons showed significantly lower scores than less cognitive impaired,  (t = -4.13***), with more positive affect in less impaired group. 2 of 3 hypotheses (67%) supported, with correlations >.3 | A | *Internal consistency:*  α = .55, no evidence of unidimensionality | D |
|  |  |  |  |  |  | *IRR*: κ = “pleasure” .62  “anger” .66  “anxiety” .28  “sadness” .65  “interest” 1.00  “tranquility” .65 | A |
|  |  |  |  |  |  | *Measurement error:* % agreement “pleasure” 92%, “anger” 97%, “anxiety” 82%, “sadness” 89%, “interest“ 100%, and  “tranquility” 89% agreement | A |
| **ODAS** | | | | | | | |
| Vogelpohl and Beck (1997) | N = 91  N_o_ = not reported  83.6% women  Moderate to severe dementia  Nursing home  Maryland, USA | Literature review. Expert group (10 geriatric-specialist nurses) to establish content validity, approach unknown. Unknown relevance of verbal items, and unclear rationale for counting behaviors/scoring | D |  |  | *IRR:* κ = .68 – 1.00. *IntraRR*: κ = .97 – .100 | A |
|  |  |  |  |  |  | *Measurement error*: % agreement before coding - 80% | D |
| **DCM-8** | | | | | | | |
| Brooker and Surr (2006) | N = 39  N_o_ = 2321  58% women  Dementia, degree of dependency from low to high.  Long term care, day hospital, day care  England | DCM originally based on poorly described field work (Kitwood & Bredin, 1994). For DCM 8, a systematic revision of DCM 7 included focus groups and interviews with experts to refine and clarify content, coding, and comprehensibility. Measure quality of life or quality of care? | A | *Hypothesis testing for construct validity*: Pearson’s correlation comparing DCM 7 and 8 was 0.97 between WIB-scores and mood and engagement-scores, indicating measuring similar constructs | A |  |  |
| Villar et al., (2015) | N = 68  N_o_ = NR  66.7% women  Moderate to severe dementia  Nursing home  Spain |  |  | *Hypothesis testing for construct validity:*  No correlation with ME and level of cognitive impairment or depression. Correlation with QoL total score (r = .39), emotional well-being (r = .47), interpersonal relations (.27), and personal development (r = .39) but not material or physical well-being, self-determinations, social inclusion or rights  5 of 6 hypotheses (83%) supported | V | *Internal consistency:*  α = .55. No evidence of unidimensionality*.* | D |
|  |  |  |  |  |  | *TRR:*  Pearson correlation between WIB-scores measured three months apart was 0.37. No evidence of patients being stable in the interim period. | D |
| **PRS** | | | | | | | |
| Perrin (1997) | N = 1  N_o_ = 58  100% women  Severe dementia  Nursing home  UK | Based on review of literature, theory and earlier instruments, theory well-described pilot-testing with experts. | V | *Hypothesis testing for construct validity:* No existing instrument to investigate construct validity |  | *Measurement error:* 82% absolute agreement for total scale | V |
| Hadley  et al., (1999) | N = 2  N_o_ = 360  50% women  Severe dementia Nursing home  UK |  |  | *Hypothesis testing for construct validity:* Testing in different contexts/interventions showed adequate sensitivity | A | *Measurement error:* 99% absolute agreement for total scale before intervention | V |
| Schall et al., (2015) | N = 9  N_o_ = >600  66.7% women  Moderate to severe dementia  In-home  observations  Germany |  |  |  |  | *IRR*: κ =.78 | A |
| **ACT** | | | | | | | |
| Wood et al., (2005) | N = 7  N_o_= 2016  57.1% women  Moderate to severe dementia  Long term care |  |  |  |  | *IRR:* κ_w_ = .89, range 0.65 – 0.99 |  |
| Wood (2005) |  | Based on literature search, theory, existing scales, extensive field work in primates. | v |  |  |  |  |
| **GCWBT** | | | | | | | |
| Kinney and Rentz (2005) | N = 12  N_o_ = 184  58% women  From mild to very severe dementia  Adult day care  Ohio, USA | Based on literature search, theory, experienced clinical observers, and pilot testing (including (Rentz, 2002). | A |  |  | *IRR*: Perfect agreement for 50 % of observations, and κ = .65 for remaining observations. | A |
| Gross et al., (2015) | N = 76  N_o_ = >234  82.9% women  Late-middle to advanced stage dementia  Residential care facility  Texas, USA |  |  | *Structural validity:* Varimax rotated PCA(N=57) participants did not support the 7 domains from Kinney (2005), but the two factors *well-being* (*r* = 0.65, *p* <.001, “interest”, “sustained attention”, “pleasure”, “self-esteem” and “normalcy”) and *ill-being* (*r* = .77, *p* = <0.001, “negative affect” and “sadness”) explaining 77.93% of the variance | D | *IRR:* Pearson correlations for full-scale: r=0.63*, “interest” r=0.68*,  “sustained attention” r=0.59*,  “pleasure” r= 0.90*  “negative affect” r= 0.42(n.s),  “sadness” r=0.51*,  “self-esteem” r=0.80*,  “normalcy” r = 0.44 (n.s). | D |
| **SM-GCWBT** | | | | | | | |
| Sauer et al. (2016) | N = 38  N_o_ = NR  78.9% women  Middle to advanced stage dementia  Adult day centre and long-term care  Ohio, USA | Further development of the GCWBT-scale, building on theory, clinical expertise, pilot testing with unclear approach | D |  |  | *IRR:* κ = .80 | A |
|  |  |  |  |  |  | *Measurement error:* Pre-assessed with training until 85% agreement was met. Unknown sample size. | D |
| Lokon et al. (2019) | N = 67  N_o_ = 567  80% women  Moderate to severe dementia  Nursing facility and continuing care retirement community  Ohio, USA |  |  | *Structural validity:* PCA supported one factor of “well-being” (“social interest”, “engagement”, “pleasure”, “disengagement” reversed) and removing “ill-being” (“negative affect”, “sadness”, “confusion”). 62% of the variance of intensity and 48% of the variance of frequency explained. Unknown model fit | D | *Internal consistency:* α =  0.70 for “well-being” and .09 for “ill-being” | V |
|  |  |  |  |  |  | *Measurement error:* Pre-assessed with training until 85% agreement was met and re-calibration each semester. Unknown sample size. | D |
| **AwareCare** | | | | | | | |
| Clare et al. (2012) | N = 40  N_o_ = 120  52.5% women  Severe dementia  Care home  UK | Literature review, building on earlier scales, focus groups with staff and family caregivers. Field work and pilot testing, and further adaptation by expert panel of practitioners and researchers | A | *Hypothesis testing for construct validity:*  Investigated with Pearson’s correlations with AwareCare “Individual responsiveness index (IRI)”.  Well-being *r*= .58, *p* = <.001***  Dementia severity *r* = -.57, *p* = <.001***  Cognitive functioning *r*= .66, *p* = <.001***  Self-care *r*= -.50, *p* = <.001***  Sensory abilities *r*= -.21, *p* = >.05  Mobility *r* = -.52, *p* = <.001***  QoL (staff) *r*= -.18, *p* = >.05  QoL (family member) *r* = -.22, *p* = >.05  5 of 8 (63%) hypothesized of relationships supported | V | *IRR:*  Mean κ = >0.6 (range -0.04 – 0.87) for response categories.  “eyes flicker” 0.11  “smiles” 0.61  “frowns” 0.66  “moves head” 0.60  “nods” -0.04  “reaches” 0.72  “moves toward” 0.49  “moves away” 0.52  “single words” 0.50  “shouts/moans” 0.87  “makes eye contact” 0.47  “explores with eyes” 0.55  “mumbles” 0.74 | V |
|  |  |  |  |  |  | *TRR:*  Describes considerable variability in response-categories followed by stimuli when re-tested one week apart. Numbers not reported | I |
| **BEAM** | | | | | | | |
| Casey et al. (2014) | N = 406  N_o_ = 1034  76.1% women  Moderate to severe dementia  Long term care  Australia | Literature review, building on theory (person centered care), operationalizations from other instruments and clinical expertise | A | *Hypothesis-testing for construct validity:* Spearman’s Rank-Order correlations between BEAM-domains and agitation, depression withdrawn behavior and quality of life correlated significantly (range *r* -.14 – .45). Six relationships were < .30, 38 % hypotheses supported | A | *IRR:* Significant Spearman’s Rho ranging .24 – 1.00 except “personal care” .11, and “high agitation” .04.  “Agitation” range  -.04 – .92,  “positive behavior” range .63 – .95.  “engagement” range .47 – .95,  “affect” range 0.56 – .94,  “global contentment” .72 | D |
| **MEDLO-tool** | | | | | | | |
| de Boer et al. (2016) | N = 16  N_o_ = 56  56 % women  Moderate to severe cognitive impairment  Nursing home  Netherlands | Building on former instruments, literature review, expert-review, and pilot testing including evaluations of feasibility, testing procedures, operationalizations, and settings | A |  |  | *IRR:* κ_w_ = (emotional well-being subscales)  “mood”^2^: .50  “agitation” 0.8 | V |
|  |  |  |  |  |  | *Measurement error:* “mood” 69.4% and “agitation” 93.2% absolute agreement. | V |
| Beerens et al. (2016) | N = 115 N = 16  N_o_ = 9660  75 % women  Moderate to severe cognitive impairment  Nursing home  Netherlands |  |  | *Hypothesis testing for construct validity:* Regression showed significant relation between higher quality of life and mood-scores (6.36, p = 0.017) | A |  |  |
| **COMMUNI-CARE** | | | | | | | |
| Lopez et al. (2016) | N =16  N_o_ = 429  25% women  Moderate to severe dementia  Psychogeriatric hospital  Spain | Building on field work and theory. One person involved in development | I | *Hypothesis testing for construct validity:* Pearson’s correlation with clinician rated improvement *r* = -0.96 | D | *Internal consistency:* α = .90. No evidence of unidimensionality. | D |
|  |  |  |  |  |  | *IRR*: κ = .87 for total scale | A |
| **QUAIDEM-ILA** | | | | | | | |
| Junge et al. (2020) | N = 150  N_o_ = NR  75% women  Moderate to severe dementia  Long term care  Germany | Building on the development of the original QUALIDEM-instrument, where patients were included in development, literature search, expert discussion. Experts decided which items to include from full-scale, including statistical testing | V^3^ | *Structural validity:* PCA showed unidimensionality with one factor accounting for 62.5% of variance. No model fit reported | A | *Internal consistency:* α ranging from .88 – .93 | V |
|  |  |  |  | *Hypothesis testing for construct validity:* Mixed effect modelling correlations in univariate/multivariate models had beta coefficients <.3 for depression (-16*/-.13*) and comprehensive QoL (17*/.14*).  No significant correlation with age, gender, session of measurement, dementia severity or functional status. 4 of 6 hypotheses (67%) confirmed | A | *TRR:*  Beta coefficient 0.40 for test-retest of momentary QoL. Stability in interim period not described. | A |
| **ENGAGEMENT** | | | | | | | |
| **MPES** | | | | | | | |
| Judge et al. (2000) | N = 19  N_o_ = 264  58% women  Mild to severe dementia  Adult day care  Ohio, USA | Developed based on expert discussion, and field testing, but unclear approach | D |  |  | *Measurement error:* 90% agreement | A |
| **OME** | | | | | | | |
| Cohen-Mansfield et al. (2009) | N = 193  N_o_ = 193  78% women  Mild to severe dementia  Nursing home  Maryland, USA | Literature review and further development of a theoretical model. Pilot testing and construction of items not described | I | *Hypothesis testing for construct validity:*  Pearson’s correlation with blinded ratings of non-validated scale of involvement in activities showed 14 of 15 correlations (93%) above .3 (range *r* = .35 – .56) | D | *IRR:*  ICC .78 for engagement  ICC .65 for action-variables | V |
|  |  |  |  |  |  | *Measurement error*:  % of agreement  84% for engagement,  92% for action variables | A |
| Cohen-Mansfield et al., (2012) | N = 302  78.9 % women  Moderate to severe dementia  Nursing home  Maryland, USA |  |  | *Hypothesis testing for construct validity:*  Spearman’s correlation with duration, attention, and attitude with positive affects (“pleasure” .32, .34, .41; “interest” .58, .60, .53) and not related to negative affect. 9 of 9 hypotheses (100%) supported. | A |  |  |
| **MIDAS** | | | | | | |  |
| McDermott et al. (2015) | N = 53  England | Pilot testing, qualitative focus groups including people with dementia, family caregivers, professional caregivers, and music therapists. Relevance of momentary effect high, but relevance of staff-ratings unclear due to unspecified observation-periods | V |  |  |  |  |
| McDermott et al. (2014) | N = 19  N_o_ = 629  74% women  People with dementia  Long term care  England |  |  | *Structural validity:*  PCA of N=629 observations supporting one factor explaining 89 % of the variance, and  PCA for the N=19 individuals (mean scores)  91 % of the variance accounted for by one factor. (range .844 – .947)** | D | *Internal consistency:*  α = .97 | V |
|  |  |  |  | *Hypothesis testing for construct validity:*  Spearman’s rank correlation with QoL *r _s_*= .48** | V | *IRR:*  ICC for therapist ratings  “interest” .79*  “response” .79*  “initiation” .77*  “involvement” .82*  “enjoyment” .79*  ICC for staff-ratings  “interest” .13  “response” .19  “initiation” .35**  “involvement” .36**  “enjoyment” .21 | V |
|  |  |  |  |  |  | *TRR:*  Therapist ratings:  “interest” 0.57  “response” 0.50  “initiation” 0.67  “involvement” 0.61  “enjoyment” 0.61 | V |
| **VC-IOE** | | | | | | | |
| Jones et al. (2015) | N = NR  N_o_ = NR  Gender NR  Population NR  Long-term care  Australia | Secured by literature review, building on earlier instruments, and referring to vaguely described qualitative field work with experts and family caregivers | I | *Hypothesis testing for construct validity:* Describes comparisons with instruments, but analysis/result not reported | I | *Measurement error:* % agreement  between raters 95.25%,  intra-rater 95 % | D |
| **EPWDS** | | | | | | | |
| Jones et al. (2018) | N = 131  N_o_ = 131  73% women  Mild to severe dementia  Long-term care  Australia | Building on earlier instruments and VC-IOE, literature review, surveying experts (N=15) to establish consensus through a Delphi procedure and piloting the scale in an intervention study (Moyle et al., 2017) | D | *Hypothesis testing for construct validity:* Pearson’s correlation with apathy scale (N=27), 12 of 12 (100%) correlations supporting construct validity (range *r* = .63 – .82) | A | *Internal consistency:*  α = .94. No evidence of unidimensionality | D |
|  |  |  |  |  |  | *IRR:* ICC .75 – .96 on single item scores  ICC .84 – .95 for subscale-scores.  Engagement .97 | A |
|  |  |  |  |  |  | *TRR:* Pearson’s correlations for individual items range 0.80 – 0.96 and total subscales range 0.86 – 0.95 | D |
| **ELICSE/EMODEB** | | | | | | | |
| Perugia et al. (2018) | N = 14  N_o_ = 42  Gender not reported  Mild to moderate dementia  Nursing home  Spain | Literature review, thorough development of ethogram, involvement of experts | V | *Hypothesis testing for construct validity*:  Using ELICSE to construct the model EMODEB, SEM shows excellent model fit (CFI = 1.00, RMSEA = 0.000) and supporting 9 of 10 hypotheses (90%) | D | *IRR*: κ = .78 for cognitive stimulation, .74 for robot-based play, head-behaviors .76 and .70, torso-behaviors .74 and 0.73, arms/hands .63 and .71. | A |
| Perugia et al., (2020) | N = 14  N_o_ = 42  Gender not reported  Mild to moderate dementia  Nursing home  Spain |  |  | *Hypothesis testing for construct validity*: Using aggregated behaviors suggested in EMODEB for Spearman’s correlations with similar instruments. Attention and valence from ELICSE correlated *r_s =_* >.3 with engagement and affect in 9 of 10 hypotheses (90%), supporting the construct validity of ELICSE | A |  |  |
| **MTED** | | | | | | | |
| Tan et al. (2019) | N = 62  N_o_ = 120  48% women  Mild to moderate dementia  Acute hospital dementia unit  Singapore | Literature review, qualitative field work, pilot-testing and revising the scale, and interview with multidisciplinary experts | A | *Structural validity:*  PCA showed one factor accounting for 64,5 % of variance. Model fit not reported. | D | *Internal consistency:*  α = .87 | A |
|  |  |  |  | *Hypothesis testing for construct validity*:  Spearman’s rank correlation with similar scales. Relevant subdomains from three similar scales correlated with four subscales and total scale (*r_s_* ranging .78 – .90) Dementia severity not correlated with score. 6 of 6 hypotheses (100%) supported | A | *IRR:*  ICC total scale .96 | A |

Abbreviations: N = number of participants in study. N_o_= number of total observations in study. RoB-ratings V = very good, A = Adequate, D= Doubtful, I = Inadequate, NA = not applicable, NE=Not evaluated. PCA = Principal component analysis, an exploratory factor analysis approach. IRR = Inter-rater reliability. IntraRR= Intra-rater reliability. TRR = Test-retest reliability for two different time-points. Re-coding of the same video by the same coder at two different time points is defined here as intra-rater reliability. ICC= Intra-class correlation. SEM = Structural equational modelling. α = Cronbach’s alpha. κ = Cohen’s kappa. Κ_w_ = weighted kappa. ^1^More data for original scale or generic version exists but is not directly transferable to this context or this version of the scale. ^2^If rarely observed, the κ_w_ may be deflated due very few disagreements, which was the case for the 5.5% “mood-disagreements”. ^3^ Content validity for QUALIDEM-ILA is partly based on development of original QUALIDEM

**Table S3** Feasibility, interpretability and use

| **Procedure and administration** | **Training and settings** | **Requirements (Copyright, equipment, cost, certification, and availability)** | **Measurement level, scoring, and distribution** | **Interpretation, sensitivity, and use in clinical contexts** |
| --- | --- | --- | --- | --- |
| **EMOTIONS** | | | | |
| **FACS/EMFACS** (Asplund et al., 1991) | | | | |
| Observer code video-recordings of the face. Repeated event recording. Coders count numbers and duration of action units per time frame (less than 1 second). 24-100 minutes coding per minute (Cohn et al., 2007; Stöckli et al., 2018) | Standardization requires 50 – 100 hours of training.  Setting requires close observation of face. Angle of face max 45 degrees turned from camera. Glasses and facial hair may interfere with coding.  Mainly a research tool | Copyrighted, certification required. Cost of manual $350, and exam $50.  Computer-assisted coding recommended. | Nominal scale, in dementia research, total score is mainly given in (relative) frequencies: presence of action units aggregated over time.  Infrequency of negative emotions (Asplund et al., 1995) | Higher frequency = more emotion present. Lack of data from clinical populations in general(Cohn et al., 2007).  Statistically sensitive for changes in mild to moderate dementia (Seidl et al., 2012), and probably sensitive to detect happiness in severe dementia (Asplund et al., 1995).  Clinical utility affected by less facial action in people with (severe) dementia |
| **MAX** (C Magai et al., 1996) | | | | |
| Observers code during direct or video observation. Repeated event recording. For each second, presence or absence of facial expression is rated. Carol Magai et al. (2002) used three coders simultaneously. | Training of unknown duration until at least 80 % agreement with MAX master code (Izard, 1979) or training tapes with facial expressions of older adults (Malatesta-Magai et al., 1992)  Setting adapted to obtain close observations of face in proper angle.  Mainly a research tool. | Copyrighted, Izards training tapes and manual (Izard, 1995) and formal training not readily available. Hand-held recorder eases coding (Magai et al., 1996). | Nominal items, in dementia research, emotions have been aggregated into continuous frequency scores of positive (joy) and negative affect (contempt, disgust, fear, sadness, anger, and shame). Items may be skewed (Magai et al., 2002), fear and disgust infrequent (Magai et al., 1996). | Mean and SD for different levels of cognitive impairment reported, with reduction of scores of joy in late stage dementia(Magai et al., 1996).  May detect clinically relevant effect sizes in hypotheses testing in the positive emotions (Magai et al., 2002)  Clinical utility lower for persons with severe dementia? |
| **OERS** (M. P. Lawton et al., 1999) | | | | |
| Rate 5-minute intervals in direct or video-recorded observations. Repeated coding of the (estimated) duration | Standardization through training with expert supervision and corrective feedback of 35-40 independent ratings (Lawton et al., 1996) Applicable in numerous (care) settings and context such as mealtime, morning care, down time, and activity time (Lawton et al., 1999).  Applicable in research and care settings | Freely available at <https://www.abramsoncenter.org/media/1199/oberved-emotion-rating-scale.pdf>  Pen and paper or handheld recorder | Ordinal items. Each items’ estimated duration is rated on a five-level ordinal scale (negative codes reversed), giving a total affect-score for each observation. In general, frequency of negative emotions skewed. Floor effect of anger (Lawton et al., 1999). | Means and SDs reported for each emotion (Lawton et al., 1996) and in different contexts (Lawton et al., 1999).  May statistically detect changes, but mostly when scores are aggregated to positive and negative emotions (i.e. Hammar et al., 2011)  Investigating and comparing present emotions during different contexts may define suitable time points for interventions. |
| **AER**(Snyder et al., 1998) | | | | |
| 5-10 minutes of direct observations. Time-sample or interval recordings, rating presence or absence of affect-indicators | Standardization required training and supervision for 2 hours in original publication (Snyder et al., 1998). 90 items, may be overwhelming to remember after 5-10 minutes of observation? Applicable in research and care settings | Unknown copyright. Pen and paper.  Scale not available in original publication. | Nominal items, scores treated as continuous in analysis. Range = 0 – 90, if indicator of affect is present (reversed for negative emotions) 15 points is scored. Skewness unknown. | Higher score indicates more positive affect. Persons with more severe cognitive impairment showed lower mean scores (more positive affect) than persons with less impairment. (M = 77.9 (SD=16.5) versus M = 68.55, (SD = 20.1).  Statistical sensitivity to change demonstrated in pilot study (Snyder et al., 2001) |
| **ODAS** (Vogelpohl & Beck, 1997) | | | | |
| 10 minutes of video-recordings where time frames of 2 minutes are repeated 5 times. Behavior is coded as present or absent for each interval (time-sample recording) (Lee et al., 2013). | Unknown standardization time, but training including monthly follow up to prevent observer drift recommended.  Mainly a research tool | Full scale freely available in original publication (Vogelpohl & Beck, 1997) Computer-assisted coding recommended | Ordinal scale treated as continuous in analysis. Total score is the sum of presence/absence scores for 5 intervals. Facial positive 0-20, facial negative 0-20, vocal positive 0-45, Vocal negative 0-50, body positive 0-30, body negative 0-40. Some skewness and infrequent items. In nursing home residents, flat affect was frequent (Beck et al., 2002) | Hypothesis-testing in intervention-study could detect change in facial and bodily displays but not verbal displays (Beck et al., 2002). Aggregation of positive and negative expressions detected clinically relevant frequencies (Lee et al., 2013)  Change in scores of expressed affect following interventions suggested to guide dementia care |
| **POSITIVE EXPRESSIONS** | | | | |
| **DCM 8** (Brooker & Surr, 2006) | | | | |
| Direct observation of repeated 5 minute-intervals, standard duration 6 hours. Time-sample recording. Observer often trained regular staff. Mapping in pairs recommended | Standardization requires 3-day course and supervision.  Observations in  common areas in care-settings for people with dementia, such as long-term care, day care, and hospital care. Up to 5 persons observed simultaneously. Suggested as more feasible in care settings than research settings (Villar et al., 2015) | Copyrighted, manual available after certification as mapper.  Pen and paper.  Cost for accredited training program $1810 ([www.alz.org.sg/artc/dcm](http://www.alz.org.sg/artc/dcm)) | Ordinal scale, scores often treated as continuous. ME scores range from +5 Very happy/very absorbed to -5 Very distressed.  Limited variability of the well-being scores of earlier versions, where the majority are scored as +1 and less than 3 % scored as -3, -5, and +5 (Sloane et al., 2007). Some improvement in DCM-8, but problem still present (Brooker & Surr, 2006). DCM may tap dependency as well as well-being and should be controlled for in analyses (Brooker & Surr, 2006; Cooke & Chaudhury, 2013) | Higher scores indicate more well-being.  Low sensitivity to change due to low variability in scores? Lower correlation for the +1 score between DCM 7 and 8 (*r* = 0.71) support increased variability of the ME-scores with new decision rules (Brooker & Surr, 2006).  Clinically important change not described, but changes in ME-scores ≥ .7 suggested to achieve statistical significance (Brooker, 2005)  An individual or group-level well-being profile may be calculated based on percentage of presence, or averaged sums |
| **PRS** (Perrin, 1997) | | | | |
| Direct observation or video-recordings, using time-sample recording. Counting presence or absence of 10 behavioral categories for repeated one-minute intervals. Categories scored twice and summarized per interval (20 seconds observation, 10 seconds scoring) | Amount of training to achieve standardization not reported. Observations of persons with severe dementia during interactions  Mainly a research tool | Full scale available in original article (Perrin, 1997).  Pen and paper. | Dichotomized scoring of nominal items, 1 = present and 0 = absent (range 0-20). For the total observation period a ratio-score is given (present behaviors/total number of time frames x 100).  Floor effect in emotional responses (“fear” in particular) in baseline-phases (Algar et al., 2016) | Higher score interpreted as increased well-being.  Created to be sensitive to change in persons with severe dementia.  Ability to detect statistically significant change demonstrated (Hadley et al., 1999; Schall et al., 2015)  Ratio in different settings may be compared to detect settings with more well-being |
| **ACT** (Wood, 2005) | | | | |
| Direct or video recordings of mutually exclusive categories for all domains. 10 minute observations per participant, consecutively repeated. Labor intensive, and maximum 4 hours repeated observations recommended to prevent observer’s fatigue | Training to achieve standardization is described as extensive (Wood et al., 2009). Used for public areas of long-term care facility.  May be customized to different environmental settings, making the instrument flexible | Freely available in original article. Pen and paper, handheld recorder or computerized coding of videos | Originally ordinal items, interval recording of events (Wood et al., 2005). Time-use may be coded as continuous (duration; Lassell et al., 2021) scores converted to relative frequencies.    Problem behaviors were rare, and no affect was present 39% of the time. | A range of the person’s functional capacity may be calculated, consisting of co-occurring positive behavior and positive affect (Wood et al., 2005), giving a level to compare interventions with.  Patterns of activity and correlates in long term care reported (Wood et al., 2005). Defined clinically significant activities when associated with QoL-indicators (Wood et al., 2009)  Sensitivity for detecting changes statistically in different contexts provided for interest and pleasure (Lassell et al., 2021) and the other domains except negative affect (Wood et al., 2005) |
| **GCWBT**(Kinney & Rentz, 2005) | | | | |
| Direct observation, scoring level of well-being indicators after (repeated) 10 minutes of observation. Gives an estimate of the duration of each indicator. | Extensive (unspecified) training required to achieve standardization.  Up to three persons observed simultaneously. Feasible settings are art interventions and other activity-settings in adult day care, assisted living, and long-term care | Full scale available in original article (Rentz, 2002).  Pen and paper | Ordinal scale transformed to continuous scale in analysis. Rating 19 indicators of well-being within the seven domains. Likert scales (0-4) from never to always (range 0 – 76)  Infrequent observations of items related to “negative affect” | Item specific frequencies or mean normalized scores reported (Kinney & Rentz, 2005; Rentz, 2002).  Statistical detection of change in positive items only |
| **SM-GCWBT** (Sauer et al., 2016) | | | | |
| Direct observation or video recordings, scoring of indicators after (repeated) 5 minute observations. Approach resembles an estimate of duration recording. | 11 hours of training gave acceptable standardization. Using video-recordings provides better agreement.  Feasible in research and care settings: art/music therapy, intergenerational creative art activity, creative staff activity, non-creative activity, and no activity for people with dementia (Lokon et al., 2019) | Full scale available in original article (Sauer et al., 2016).  Pen and paper, tablets with software may ease data-collection | Ordinal scale, rating 25 different indicators of well-being and ill-being for level of frequency (infrequent, some of the time or most of the time) and intensity (low, moderate or high)  Calculating percentage of time the specific level of intensity or frequency was observed enables parametric testing  Ill-being items very infrequent and suggested to be removed (Lokon et al., 2019) | Mean frequency and intensity (%) with SDs for well-being during different activities in the dementia populations provided (Lokon et al., 2019).  Statistical sensitivity for detecting change in positive items demonstrated (Lokon et al., 2019; Sauer et al., 2016) |
| **AwareCare** (Clare et al., 2012) | | | | |
| Direct observation for 30 minutes. Event recording where present stimuli is recorded followed by the response to the stimuli. | Required training for standardization not reported. Some operationalizations of stimuli/response suffered from low comprehensibility  Feasible for research and care settings. Applicable settings are public areas in long term care facilities, during wakefulness. Pre-defined stimuli were not personal, and personalizing stimuli will increase feasibility | Full scale provided upon request to corresponding author (Clare et al., 2012).  Pen and paper | Each response category is dichotomously coded as present or absent. A Stimuli -Responsiveness Index is calculated on group and individual level based on a ratio (stimuli followed by response/number of occurrences of stimuli).  Steps to remove infrequent items during development of scale prevented floor and ceiling-effects. | Mean Stimulus Responsiveness Index was 2.65 (SD=0.94, range 0.97-4.49).    The instrument detected signs of awareness in all participants (Clare et al., 2012). Statistical sensitivity as AwareCare score predicted QoL-score (Clare, 2014)  The Stimulus Responsiveness Index enables assessing individual processes and comparisons on group-levels |
| **BEAM** (Casey et al., 2014) | | | | |
| Duration recording during direct observation. Mutually exclusive categories are coded for 10-minute intervals | Required training for standardization not reported. 4 persons may be observed concurrently. Tested in different levels of care. Feasible for assessing psychosocial interventions in long term care: staff-facilitated activity, unstructured free time, and mealtime | Full scale described in publication (Casey et al., 2014)  Tablet with BEAM-coding system required | Mutually exclusive categorical domains scored in terms of duration, and interaction-items scored in terms of frequencies. Global contentment rated on a Likert scale from 1 (ill-being) to 7 (well-being)  Duration-based items were not normally distributed, and some behaviors were naturally infrequent | Relative frequencies of the different domains provided (Casey et al., 2014)  Statistically sensitive to change in items “happiness” and “agitation” (Low et al., 2014) |
| **MEDLO-tool** (de Boer et al., 2016) | | | | |
| Direct observation for one minute followed by rating per resident. Time-sample recording.  Easily administered, observers fatigue accounted for | Standardization acquired through 20 hours of training including supervised practice and evaluation.  Feasible for assessing aspects of daily living in long term care, tested in different settings and activities | Tablet with MEDLO-tool app installed. App available upon request, manual freely available online (de Boer et al., 2016) | Ordinal scale with level of items in emotional well-being scored:  Mood – 1 (low) to 7 (high)  Agitation – 1(low) to 5 (high)  Low variability in the mood score, mostly rated in the middle. Skewed distribution with agitation very infrequent (Beerens et al., 2016; de Boer et al., 2016) | Mean scores of “mood” during different activities described (Beerens et al., 2018)  Statistically sensitive to changes in mood and its relation to “activities of daily living” (Beerens et al., 2016; Beerens et al., 2018) |
| **COMMUNI-CARE** (Lopez et al., 2016) | | | | |
| Direct observation by independent observer or the facilitator of the intervention. 10-minute intervals repeated three times. Gives estimates of events. | Required training for standardization not reported.  Evaluates Snoezelen interventions, but described as transferable to other psychosocial interventions | Copyright not reported.  Pen and paper  Operationalizations of scale not reported in publication | Ordinal scale where the sum is a composite score used as a continuous outcome in analyses. Level of each subscale is rated (range 1 – 5) based on average negative, positive or indifferent behavioral responses in the observation period  Two scoring options: Global score for the session (range 5 – 25) or three intervals summarized (range 15 – 75) | Mean scores in the developmental study reported (11.70 – 12.56 of 25) for each observation-period  Clinical interpretation of scores provided, with cut-off-scores describing positive, indifferent, or negative response (Lopez et al., 2016) |
| **QUALIDEM-ILA** (Junge et al., 2020) | | | | |
| Direct observation in care settings. Scale rated before and after intervention (30 minutes). Time frame not clearly described. Scores give estimates of frequencies | Required training or achievable standardization not reported.  May be used for people with mild to severe dementia before and after interventions, in research and ordinary care | Full scale available in original QUALIDEM-publication (Ettema et al., 2007).  Pen and paper and/or tablet | Ordinal scale where the sum is a composite score used as a continuous outcome (range 0 – 24). Item levels are scored from never to frequently (range 0 – 3). | Higher scores indicate better momentary QoL. Mean composite scores were 5.4 (SD 1.2) (Junge et al., 2020) |
| **ENGAGEMENT** | | | | |
| **MPES** (Judge et al., 2000) | | | | |
| Direct observation where duration of the mutually exclusive categories are scored in 10-minutes (repeated) interval(s). Continuous time sampling | Required training for standardization not reported. Comparing regular to Montessori-based interventions | Full scale available (Orsulic-Jeras et al., 2000)  Stop-watch, pen, and paper | Nominal items with duration in seconds, range of scoring from 0-600 per observation interval. Scores used as frequency (percentage) item was present, or durations in seconds. “Non-engagement” and “Self-engagement” were infrequent (Orsulic-Jeras et al., 2000) | Means and SDs provided for the different items (Orsulic-Jeras et al., 2000).  Statistical sensitivity to detect changes between groups (Lee et al., 2007; Orsulic-Jeras et al., 2000) |
| **OME** (Cohen-Mansfield et al., 2009) | | | | |
| Direct observation during presentation of stimuli in time frames of 15 minutes. Subscales scored once during each observation. Combines different sampling methods | Standardization among research assistants described but amount of training not reported.  May be used to assess 24 different stimuli, where using personalized stimuli is optional and increase feasibility | All items and scoring options described in the original article (Cohen-Mansfield et al., 2009).  Handheld computer with software of unknown  availability | All items treated as ordinal (Cohen-Mansfield et al., 2009)  Rate of refusal - yes/no,  Duration recorded in seconds (but transformed to ordinal level)  Attention – 4 point scale of level  Attitude – 7 point scale  Activity – 4 point scale of time spent | Median durations and effect-size estimates (Odds Ratios with CI) available.  Statistical sensitivity to detect change demonstrated. Reports a need for further work to increase clinical utility (Cohen-Mansfield et al., 2012; Cohen-Mansfield et al., 2011) |
| **MIDAS** (McDermott et al., 2014) | | | | |
| Direct observation completed four times.  1. Before intervention (average impression of the person that day)  2. The first five minutes of music therapy  3. The five most significant minutes during music therapy  4. “A few” hours after intervention (average impression after intervention)  Observation time for staff is “today”, but for music therapist ratings are for 5-minute intervals | Easily standardized for music therapist, issues regarding standardization for staff  Feasible for observations of music therapy in care-settings where staff-ratings may occur before and after interventions. Several people may be observed simultaneously | Pen and paper. Scale freely available at <https://www.musictherapy.aau.dk/midas/midas-forms/> (including translations to different languages) | Continuous scale. The visual analogue items range from 0 – 100mm, where 100 represents the highest possible level of the items for the specific individual, taking their degenerative disease in to account. Total score range 0 -500 per observation. Some skewness reported that could be normalized with transformation | Mean scores are 238.87 (SD =136.8) of 500 (McDermott et al., 2014).  Statistical sensitivity to detect change of momentary assessments from before to after music therapy demonstrated for all VAS-items (Garrido et al., 2020) |
| **VC-IOE** (Jones et al., 2015) | | | | |
| Video-recorded observations. Duration of each engagement behavior is recorded. Continuous time sampling. 3-4 minutes of coding per observational minute. Videos in project lasted 30-45 minutes | Required training for standardization not reported.  Designed to be used for research in care-settings for pre-post intervention-studies. Tested in settings with persons with dementia interacting with social robots in in long-term care  Mainly a research tool | Full scale freely available in original article (Jones et al., 2015).  Computerized coding such as Noldus Observer® recommended | Nominal items, duration in seconds scored for each mutually exclusive behavior category creates a continuous score.  Distribution not reported | Total score is described as a meaningful interpretation of engagement-level.  Statistical sensitivity and clinical interpretation uncertain |
| **EPWDS** (Jones et al., 2018) | | | | |
| Direct or video-recorded observations of 10 minutes where items are scored after the interval. Scores are estimates of engagement level in the interval | Training not reported, but standardization was acquired when using experienced raters.  May be used in research and acute, community and long-term care | Full scale freely available in original article (Jones et al., 2018).  Pen and paper | Ordinal scale. Likert scoring from 1 (strongly disagree) to 5 (strongly agree). Total sum (negative reversed) treated as continuous score (range 10 – 50) where disengagement is reverse scored. | Higher composite scores indicate higher level of engagement.  Statistical sensitivity to change demonstrated for visual, behavioral, social, and composite score, but found affective engagement not providing good estimates of frequency (Feng et al., 2020)  Suggest creating an individual baseline to interpret individual clinical change |
| **ELICSE/EMODEB** (Perugia et al., 2018) | | | | |
| Video-observations where durations of mutually exclusive behaviors and their positive/negative gestures are coded. Continuous time sampling. | Training not reported, but standardization documented.  Developed for robot and game-based play, applicable in research settings where activity does not entail physical effort and the activity is handling artifacts | Coding scheme available as supplementary material in original article. Computer-assisted coding required | Nominal items, duration in seconds scored for each mutually exclusive behavior category creates a continuous score.  A total engagement-score for each body-part based on the duration of behavior is given, ranging from -100 (max. disengagement) to 100(max engagement)  Some behaviors were naturally infrequent, but this was not reported as a problem in the EMODEB-model | Frequencies reported, and structure of items tested in a model (Perugia et al., 2018).  Interpretation suggested to be investigated further, but score aims to be traced back to an engagement level |
| **MTED** (Tan et al., 2019) | | | | |
| Direct observation, independent observer or music therapist estimate level of each item once. Time frame is one music therapy session (approximately 30 minutes) | Required training for standardization not reported.  Used for individual observations of music therapy sessions, to detect the process of music-therapy over time. Could detect engagement independent of dementia severity | Full scale freely available in original article (Tan et al., 2019)  Pen and paper | Ordinal scale. Each item scored from low to high (range 1 – 4) and summarized, giving a total score (range 4 – 20) per observation | Higher score indicates more engagement. Mean score is 13.02 (SD=3.5) of 20.  Statistical sensitivity uncertain |

**Appendix A – Search strategy**

| **Search strategy** |
| --- |
| **MEDLINE, EMBASE and PsycInfo (Ovid):**  (“dementia*” OR “alzheimer*”) AND  (“wellbeing” OR “well-being” OR “positive feeling*” OR “positive emotion*” OR (emotion* ADJ3 express*) OR “SWB”) AND  (Observ* or (prox* adj2 rate*) or “experience sampling” or ESM or “ecological* momentary assessment*” or EMA or “ambulatory monitor*” or “event based sampling” OR “instrument*” OR “outcome scale*” OR “measure*” OR “validation stud*” OR “psychometr*” OR “reproducibility of result*” OR “reliab*” OR “unreliab*” OR “valid*” OR “coefficient*” OR “agreement*” OR (test* adj2 retest*) OR “inter-rater*” OR “interrater*” OR “intrarater*” OR “intra-rater*” OR “inter-observer*” OR “interobserver*” OR “inter-examiner*” OR “interexaminer*” OR (factor* ADJ2 analys*) OR clinimetr* OR clinometr* OR (outcome adj2 ‎assessment*) OR (outcome* adj2 ‎measure*‎) OR ‎(observer* adj2 variation*) ‎OR reproducib* OR ‎homogeneity OR homogeneous ‎‎OR ‎‎“internal consistency” OR ‎agreement* OR precision OR ‎imprecision ‎OR “precise ‎values” ‎OR “test retest” OR ‎stability ‎OR ‎intertechnician* OR inter-technician* ‎OR intratechnician ‎OR intra-‎technician ‎OR interexaminer* OR ‎inter-examiner* OR intraexaminer* ‎OR intra-examiner* OR ‎interassay ‎OR ‎inter-assay OR intraassay OR ‎intra-assay OR ‎interindividual OR ‎inter-individual ‎OR ‎intraindividual ‎OR intra-individual OR ‎interparticipant* OR inter-participant* ‎OR intraparticipant* ‎OR ‎intra-‎participant* OR ‎kappa OR kappa’s ‎OR kappas OR repeatab*‎ OR ‎generaliza* OR generalisa* OR ‎concordance* OR discriminative OR ‎‎“known group” OR dimension* OR ‎subscale* ‎OR “‎item discriminant*” ‎OR “interscale ‎correlation*” OR error* OR “‎individual variability” OR “standard ‎error* of ‎measurement*” OR sensitiv* ‎OR responsive* OR “meaningful ‎change*” OR ‎‎“ceiling effect*” OR ‎‎“‎floor effect*” OR “Item response ‎model*” OR IRT OR Rasch OR “‎Differential ‎item functioning” OR ‎DIF OR ‎‎“computer adaptive testing‎” OR “item bank” OR “cross-cultural ‎‎equivalence”‎ OR ‎“cronbach* ‎alpha*” OR (replicab* adj2 measure*) ‎OR (replicab* adj2 finding*) OR (‎replicab* adj2 ‎result*) OR ‎‎(replicab* adj2 ‎test*) OR “repeated measure*” OR ‎‎“repeated finding*” OR “repeated ‎result*” OR ‎‎“repeated test*” OR (‎item* adj2 correlation*) ‎OR (item* ‎adj2 selection*) OR (item* adj2 reduction*) ‎OR “intraclass ‎correlation*” OR “multitrait scaling ‎analys*” OR (uncertaint* adj2 measur*) ‎OR (variabilit* ‎adj2 analys*) OR (‎variabilit* adj2 value*) OR (minimal* ‎adj2 change) OR (minimal* adj2 difference*) ‎OR (clinical* adj2 change*) OR (clinical* adj2 ‎difference*) OR (small* adj2 change*) OR (small* adj2 ‎difference*)) OR error* |
| **Web of Science:**  **TOPIC:** ((“dementia*” or “Alzheimer*”)) *AND*  **TOPIC:** (("wellbeing" OR "well-being" OR "positive feeling*" OR "positive emotion*" OR (emotion* ADJ3 express*) OR "SWB")) *AND*  **TOPIC:** (("observ*" or "proxy-rated" or "instrument*" OR "outcome scale*" OR "measure*" OR "validation stud*" OR "psychometr*" OR "reproducibility of result*" OR "reliab*" OR "unreliab*" OR "valid*" OR "coefficient*" OR "agreement*" OR (test* ADJ2 retest*) OR "inter-rater*" OR "interrater*" OR "intrarater*" OR "intra-rater*" OR "inter-observer*" OR "interobserver*" OR "inter-examiner*" OR "interexaminer*" OR (factor ADJ2 analys*) )) OR clinimetr* OR clinometr* OR (outcome ADJ2 ‎assessment‎‎*) OR (outcome* ADJ2 ‎measure*‎) OR ‎(observer* ADJ2 variation*) ‎OR reproducib* OR ‎homogeneity OR homogeneous ‎‎OR ‎‎“internal consistency” OR ‎agreement* OR precision OR ‎imprecision ‎OR “precise ‎values” ‎OR “test retest” OR ‎stability ‎OR ‎intertechnician* OR inter-technician* ‎OR intratechnician ‎OR intra-‎technician ‎OR interexaminer* OR ‎inter-examiner* OR intraexaminer* ‎OR intra-examiner* OR ‎interassay ‎OR ‎inter-assay OR intraassay OR ‎intra-assay OR ‎interindividual OR ‎inter-individual ‎OR ‎intraindividual ‎OR intra-individual OR ‎interparticipant* OR inter-participant* ‎OR intraparticipant* ‎OR ‎intra-‎participant* OR ‎kappa OR kappa’s ‎OR kappas OR repeatab*‎ OR ‎generaliza* OR generalisa* OR ‎concordance* OR discriminative OR ‎‎“known group” OR dimension* OR ‎subscale* ‎OR “‎item discriminant*” ‎OR “interscale ‎correlation*” OR error* OR “‎individual variability” OR “standard ‎error* of ‎measurement*” OR sensitiv* ‎OR responsive* OR “meaningful ‎change*” OR ‎‎“ceiling effect*” OR ‎‎“‎floor effect*” OR “Item response ‎model*” OR IRT OR Rasch OR “‎Differential ‎item functioning” OR ‎DIF OR ‎‎“computer adaptive testing‎” OR “item bank” OR “cross-cultural ‎‎equivalence”‎ OR ‎“cronbach* ‎alpha*” OR (replicab* ADJ2 measure*) ‎OR (replicab* ADJ2 finding*) OR (‎replicab* ADJ2 ‎result*) OR ‎‎(replicab* ADJ2 ‎test*) OR “repeated measure*” OR ‎‎“repeated finding*” OR “repeated ‎result*” OR ‎‎“repeated test*” OR (‎item* ADJ2 correlation*) ‎OR (item* ‎ADJ2 selection*) OR (item* ADJ2 reduction*) ‎OR “intraclass ‎correlation*” OR “multitrait scaling ‎analys*” OR (uncertaint* ADJ2 measur*) ‎OR (variabilit* ‎ADJ2 analys*) OR (‎variabilit* ADJ2 value*) OR (minimal* ‎ADJ2 change) OR (minimal* ADJ2 difference*) ‎OR (clinical* ADJ2 change*) OR (clinical* ADJ2 ‎difference*) OR (small* ADJ2 change*) OR (small* ADJ2 ‎difference*)) OR error* |
| **CINAHL:**  ( (dementia* or alzheimer*) ) AND ( (wellbeing OR "well-being" OR "positive feeling*" OR "positive emotion*" OR (emotion* N2 express*) OR SWB) ) AND ( (observ* OR (prox* N1 rate*) OR "experience sampling" OR ESM OR "ecological* momentary assessment*" OR EMA OR "ambulatory monitor*" OR "event based sampling" OR instrument* OR "outcome scale*" OR measure* OR "validation stud*" OR psychometr* OR "reproducibility of result*" OR reliab* OR unreliab* OR valid* OR coefficient* OR agreement* OR (test* N1 retest*) OR "inter-rater*" OR interrater* OR intrarater* OR "intra-rater*" OR "inter-observer*" OR interobserver* OR "inter-examiner*" OR interexaminer* OR (factor* N1 analys*) OR clinimetr* OR clinometr* OR (outcome N1 assessment*) OR (outcome* N1 measure*) OR (observer N1 variation*) OR reproducib* OR homogeneity OR homogenous OR "internal consistency" OR agreement* OR precision OR imprecision OR "precise values" OR "test retest" OR stability OR "internal consistency" OR "inter-technician*" OR intertechnician* OR intratechnician* OR "intra-technician*" OR interexaminer* OR "inter-examiner*" OR intraexaminer* OR "intra-examiner*" OR interassay OR "inter-assay" OR intraassay OR "intra-assay" OR interindividual OR "inter-individual" OR intraindividual OR "intra-individual" OR interparticipant* OR "inter-participant*" OR intraparticipant* OR "intra-participant*" OR kappa* OR repeatab* OR generaliza* OR generalisa* OR concordance* OR discriminative OR “known group” OR dimension* OR ‎subscale* ‎OR “‎item discriminant*” ‎OR “interscale ‎correlation*” OR error* OR “‎individual variability” OR “standard ‎error* of ‎measurement*” OR sensitiv* ‎OR responsive* OR “meaningful ‎change*” OR ‎‎“ceiling effect*” OR ‎‎“‎floor effect*” OR “Item response ‎model*” OR IRT OR Rasch OR “‎Differential ‎item functioning” OR ‎DIF OR ‎‎“computer adaptive testing‎” OR “item bank” OR “cross-cultural ‎‎equivalence”‎ OR ‎“cronbach* ‎alpha*” OR (replicab* N1 measure*) ‎OR (replicab* N1 finding*) OR (‎replicab* N1 ‎result*) OR ‎‎(replicab* N1 ‎test*) OR “repeated measure*” OR ‎‎“repeated finding*” OR “repeated ‎result*” OR ‎‎“repeated test*” OR (‎item* N1 correlation*) ‎OR (item* ‎N1 selection*) OR (item* N1 reduction*) ‎OR “intraclass ‎correlation*” OR “multitrait scaling ‎analys*” OR (uncertaint* N1 measur*) ‎OR (variabilit* ‎N1 analys*) OR (‎variabilit* N1 value*) OR (minimal* ‎N1 change) OR (minimal* N1 difference*) ‎OR (clinical* N1 change*) OR (clinical* N1 ‎difference*) OR (small* N1 change*) OR (small* N1 ‎difference*)) OR error* |
| **ProQuest:**  noft((dementia* OR alzheimer*)) AND  noft((wellbeing OR “well-being” OR “positive feeling*” OR “positive emotion*” OR (emotion* NEAR/3 express*) OR SWB)) AND  noft((observ* OR (prox* NEAR/2 rate*) OR “experience sampling” OR ESM OR “ecological* momentary assessment*” OR EMA OR “ambulatory monitor*” OR “event based sampling” OR state OR momentar* OR “continuous time sampl*” OR “time sampl*” OR instrument* OR “outcome scale*” OR measure* OR “validation stud*” OR psychometr* OR “reproducibility of result*” OR reliab* OR unreliab* OR valid* OR coefficient* OR agreement* OR (test* NEAR/2 retest*) OR “inter-rater*” OR interrater* OR intrarater* OR “intra-rater*” OR “inter-observer*” OR interobserver* OR “inter-examiner*” OR interexaminer* OR (factor* NEAR/2 analys*) OR clinimetr* OR clinometr* OR (outcome NEAR/2 ‎assessment*) OR (outcome* NEAR/2 ‎measure*‎) OR (observer NEAR/2 variation*) OR reproducib* OR homogeneity OR homogenous OR “internal consistency” OR agreement* OR precision OR imprecision OR “precise values” OR “test retest” OR stability OR “internal consistency” OR “inter-technician*” OR intertechnician* OR intratechnician* OR “intra-technician*” OR interexaminer* OR “inter-examiner*” OR intraexaminer* OR “intra-examiner*” OR interassay OR “inter-assay” OR intraassay OR “intra-assay” OR interindividual OR “inter-individual” OR ‎intraindividual ‎OR “intra-individual” OR ‎interparticipant* OR “inter-participant*” ‎OR intraparticipant* ‎OR “‎intra-‎participant*” OR ‎kappa* ‎OR repeatab*‎ OR ‎generaliza* OR generalisa* OR concordance* OR discriminative OR “known group” OR dimension* OR subscale* OR “item discriminant*” OR “interscale correlation*” OR “individual variability” OR “standard error* of measurement*” OR alzheimer* OR responsive* OR “meaningful change*” OR “ceiling effect*” OR “floor effect*” OR “Item response model*” OR IRT OR Rasch OR “Differential item functioning” OR DIF OR “computer adaptive testing” OR “item bank” OR “cross-cultural equivalence” OR “alzheime* alpha*” OR alzheim* NEAR/2 measure*) OR (alzheim* NEAR/2 finding*) OR (alzheim* NEAR/2 result*) OR (alzheim* NEAR/2 test*) OR “repeated measure*” OR “repeated finding*” OR “repeated result*” OR “repeated test*” OR (item* NEAR/2 correlation*) OR (item* NEAR/2 selection*) OR (item* NEAR/2 reduction*) OR “intraclass correlation*” OR “multitrait scaling analys*” OR (uncertaint* NEAR/2 measur*) OR alzheimer* NEAR/2 analys*) OR (alzheimer* NEAR/2 value*) OR (minimal* NEAR/2 change*) OR (minimal NEAR/2 difference*) OR (clinical* NEAR/2 change*) OR (clinical NEAR/2 difference*) OR (small* NEAR/2 change*) OR (small* NEAR/2 difference*) OR error*))  AND PEER(yes) |

1. The adapted guidelines are based on the COSMIN Risk of Bias checklist for systematic reviews of Patient-Reported Outcome Measures (Mokkink et al., 2017; Terwee et al., 2018) which is available for download on. and specific criteria for establishing content validity of observational instruments based on recommendations from Bakeman and Quera (2011) and Chorney, McMurtry, Chambers, and Bakeman (2015). The original texts are written in italics, and changes are in plain text. [↑](#footnote-ref-1)
2. Finding relevant items is based on identifying issues important to the patients and extracting relevant items from these issues (Brod, Tesler, & Christensen, 2009). For observational instruments we recognize several approaches appropriate for identifying relevant issues. The main criteria are to show adequate research rigour in the specific research approach [↑](#footnote-ref-2)
3. The difference between development studies and studies on content validity is that the latter refers to studies conducted after the final ObsROM-version. Criteria is that a new sample of participants are included (independent of the development study) [↑](#footnote-ref-3)
4. For PROMS, patients should be asked about relevance, comprehensiveness and comprehensibility of the instrument. For observational instruments, inclusion of patients is an advantage, but relevance, comprehensiveness and comprehensibility need to be evaluated at minimum by the observers actually scoring the instrument. [↑](#footnote-ref-4)
